# Synergistic Antitumoral Effect of Epigenetic Inhibitors and Gemcitabine in Pancreatic Cancer Cells

**DOI:** 10.3390/ph15070824

**Published:** 2022-07-02

**Authors:** Immacolata Maietta, Amparo Martínez-Pérez, Rosana Álvarez, Ángel R. De Lera, África González-Fernández, Rosana Simón-Vázquez

**Affiliations:** 1 CINBIO, Immunology Group, Universidade de Vigo, 36310 Vigo, Spain; immacolata.maietta@uvigo.es (I.M.); ammartinez@uvigo.es (A.M.-P.); africa@uvigo.es (Á.G.-F.); 2 Instituto de Investigación Sanitaria Galicia Sur (IIS Galicia Sur), SERGAS-UVIGO, 36312 Vigo, Spain; rar@uvigo.es (R.Á.); qolera@uvigo.es (Á.R.D.L.); 3 CINBIO, ORCHID Group, Universidade de Vigo, 36310 Vigo, Spain

**Keywords:** epigenetic enzymes, anti-tumoral drugs, 3D cell culture, RNA-Seq, Entinostat, UVI5008

## Abstract

Epigenetic modifications could drive some of the molecular events implicated in proliferation, drug resistance and metastasis of pancreatic ductal adenocarcinoma (PDAC). Thus, epigenetic enzyme inhibitors could be the key to revert those events and transform PDAC into a drug-sensitive tumor. We performed a systematic study with five different epigenetic enzyme inhibitors (1, UVI5008, MS275, psammaplin A, and BIX01294) targeting either Histone Deacetylase (HDAC) 1 or 1/4, DNA methyltransferase 3a (DNMT3a), Euchromatic histone lysine methyltransferase 2 (EHMT2), or Sirtuin 1 (SIRT1), as well as one drug that restores the p53 function (P53R3), in three different human PDAC cell lines (SKPC-1, MIA PaCa-2, and BxPC-3) using 2D and 3D cell cultures. The synergistic effect of these antitumoral drugs with gemcitabine was tested and the most efficient combinations were characterized by RNA-seq. The inhibition of HDAC1/4 (MS275), HDAC1/4/SIRT1/DNMT3a (UVI5008) or EHMT2 (BIX01294) induced a significant reduction on the cell viability, even in gemcitabine-resistance cells. The combination of UVI5008 or MS275 with gemcitabine induced a synergistic effect at low concentration and the RNA-Seq analysis revealed some synergy candidate genes as potential biomarkers. Reverting aberrant epigenetic modifications in combination with gemcitabine offers an alternative treatment for PDAC patients, with an important reduction of the therapeutic dose.

## 1. Introduction

Pancreatic ductal adenocarcinoma (PDAC) is one of the most aggressive cancers, with a 5-year overall survival of less than 8% [1]. PDAC is originated from the ductal epithelial cells that form the capillary-like duct system [2], leading to tumor growth and environmental changes, moving from a pro-inflammatory to a highly fibrotic and immune suppressive scenario [3]. Because the detection of PDAC arises often in advanced tumor stages, in many cases, patients have metastasis when diagnosed. As a result, anti-cancer therapeutics are usually not effective, showing the aggressive PDAC’s strong cytoprotective mechanisms that promote drug resistance [4].

The most frequent mutations in PDAC are limited to four proteins: KRAS (in ca. 85% of cases), TP53 (60–70%), CDKN2A (50%), and SMAD4 (~50%). However, the complex molecular oncogenic signature induced by epigenetic modifications and dysregulation of several signaling pathways, which are involved in both tumor progression and resistance, makes PDAC an undruggable tumor [5,6].

For PDAC patients with resectable lesions, the standard treatment includes surgery and chemotherapy: gemcitabine, capecitabine, or 5-fluorouracil (5-FU) [1]. In the case of metastasis, gemcitabine, FOLFIRINOX (combination of folinic acid (leucovorin), 5-FU, irinotecan, and oxaliplatin), or nab-paclitaxel, are the standard used drugs. These agents collectively interfere with DNA replication and transcription [7].

Although the available treatments can profoundly improve the prognosis of advanced pancreatic cancer, the development of chemoresistance, especially to gemcitabine, still leads to poor clinical outcomes and new effective drugs are strongly needed. To circumvent gemcitabine resistance in PDAC, many molecular-targeted agents that interact with crucial pathways for cell survival in pancreatic cancer are currently being explored. These include compounds that target enzymes implicated in epigenetic pathways as Histone Deacetylase (HDAC), DNA methyltransferase (DNMT), Euchromatic histone lysine methyltransferase 2 (EHMT2) or G9a [8], and Sirtuins (SIRT) [9]. They could represent promising new therapeutic targets for the treatment of PDAC [10], either alone or in combination with gemcitabine [11,12,13,14]. 

Following this idea, we performed a systematic study to compare the anti-tumoral effectiveness of five different epigenetic enzyme inhibitors (named 1, UVI5008, MS275, psammaplin A, and BIX01294) on three different PDAC cell lines. These compounds, including natural product psammaplin A, were designed to inhibit individual or several enzymes such as HDAC1, HDAC4, DNMT3a, SIRT, and EHMT2. The inhibition of these relevant epigenetic enzymes implicated in tumoral progression, and their potential combination with gemcitabine, make them especially interesting for PDAC therapy. In addition, we tested a molecule that restores the p53 function (P53R3) since TP53 is the second most frequent mutated gene in PDAC. Oncogenes such as RAS activate the tumor-suppressor p53 signaling protein, which in turn triggers cell cycle arrest or apoptosis. However, p53 is frequently mutated in metastatic PDAC, providing an escape mechanism to the tumor cells [15]. For that reason, restoring the p53 function could be an efficient adjuvant therapy to eradicate this complex tumor. 

Additionally, 1 is a sulphonamide analogue, sharing the benzanilide group of the HDAC inhibitor MS-275. It has shown effects as HDAC1 inhibitor on several tumoral cell lines (colon, lung, prostate), inducing an increase on the p21 levels, the acetylation of histone 3 and 4, and the arrest on the G2-M phase of the cell cycle [16].

UVI5008 is a derivative of the natural compound psammaplin A [17], which shows a broad inhibition activity, targeting HDAC1, HDAC4, SIRT1, and DNAMT3a [18]. It was found to display p53-independent tumor-selective activity *in vitro*, in leukemia patients’ blasts *ex vivo* and in mouse xenografts *in vivo* by acting directly on tumor cells, inducing TRAIL-dependent and ROS-mediated apoptosis and displaying favorable pharmacokinetics and tumor targeting [18]. In addition to the antitumor effect, this compound exerted a specific growth inhibition activity against the bacteria *Staphylococcus aureus* showing structural modifications of the cell wall as observed by SEM electromicroscopy [17]. 

MS275 (commercial name Entinostat), is a synthetic benzamide derivative histone deacetylase (HDAC) inhibitor, which selectively inhibits class I and IV HDAC enzymes. It has been shown that Entinostat enhances the inhibition induced by gemcitabine on cell proliferation, together with the induction of cell death by apoptosis in some pancreatic cancer cell lines [19]. Moreover, it is being studied in solid tumors in combination with hormone therapy and monoclonal antibodies directed against check point inhibitors (PD-1/PD-L1) [20].

Psammaplin A is a dimeric natural product that was originally isolated from an unidentified sponge, which displays antibacterial and antitumor activities by inhibition of both HDAC1 and DNMT1 [21,22]. Psammaplin A also inhibits aminopeptidase N (APN), mycothiol-S-conjugate amidase (MCA), topoisomerase II, farnesyl protein transferase, and leucine aminopeptidase. All these factors are involved in tumor progression, adhesion, proliferation, angiogenesis, and tumor invasion [23].

BIX01294 is a diazepine-quinazolinamine derivative, which specifically inhibits the euchromatic histone methyltransferase 2 (EHMT2) enzymatic activity and reduces histone H3 at lysine 9 (H3K9) dimethylation levels at the chromatin regions of several EHMT2 target genes [24,25]. EHMT2 or G9a is a histone methyl transferase that primarily catalyzes mono- and dimethylation of histone H3K9 to afford H3K9me1/me2, being overexpressed in several cancers, including head and neck squamous carcinoma, breast cancer, and aggressive ovarian and bladder cancers [26].

P53R3 is a p53 rescue compound identified from a small library using an in vitro DNA binding assay that restores sequence-specific DNA binding of the endogenously expressed p53R175H, p53R248W, and p53R273H mutants [27]. P53R3 strongly enhances the mRNA, total protein, and cell surface expression of several pro-apoptotic p53 target genes, like the death receptor 4 and 5 (DR4, DR5) and Apo2L/TRAIL receptors. P53R3 was considered to hold potential for the treatment of cancers that are resistant to the therapeutic induction of apoptosis [27].

Since there can be a variety of pancreatic tumor signatures, with different susceptibility to the conventional anti-tumoral drugs, it is important to analyze various cell types. The information provided could be crucial for a more personalized medicine, adapting the drug to the specific type of tumor. We tested those compounds on three different human tumoral pancreatic cell lines (SKPC-1, MIA Paca-2, and BxPC-3) that could represent different types of human cancer cells regarding gemcitabine resistance. These drugs were used alone or in combination with gemcitabine, looking for synergistic effects on cell viability, to find the highest anti-tumoral effect with the lowest possible dose of each of them.

For those combinations of compounds showing synergistic effects with gemcitabine in any of the cell lines tested, the differentially expressed genes respect to the untreated cells were studied by RNA-seq and compared with the individual compounds, trying to identify synergy candidate genes as potential biomarkers.

## 2. Results and Discussion

### 2.1. Effect of Epigenetic Inhibitors and P53R3 on the Viability of Three Different Human Pancreatic Cells Lines in 2D Cell Cultures

**Figure 1:**
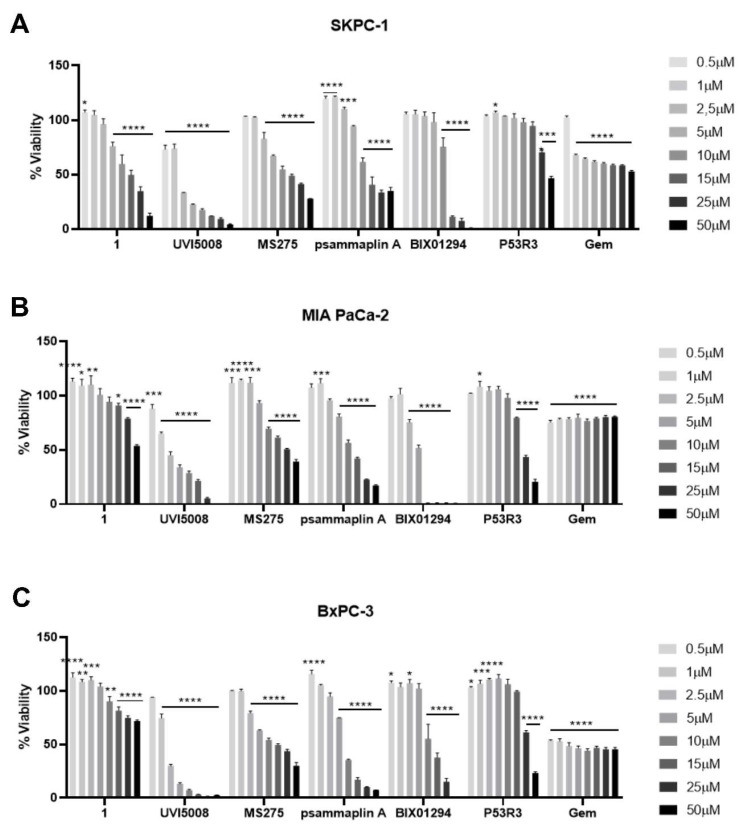
Antiproliferative effect induced by 1 (MS275 analogue), 2 (UVI5008), 3 (MS275), 4 (psammaplin A), 5 (BIX01294), 6 (P53R3), and gemcitabine (Gem) on three different pancreatic cell lines SKPC-1 (**A**), MIA PaCa-2 (**B**), and BxPC-3 (**C**) after 48 h of incubation, determined by MTS assay. Statistically significant differences compared to untreated cells were represented as: * *p* ≤ 0.05, ** *p* ≤ 0.01, *** *p* ≤ 0.001, **** *p* ≤ 0.0001.

To determine their potential antitumoral therapeutic effect, five different epigenetic inhibitors (1, UVI5008, MS275, psammaplin A, and BIX01294) and a molecule that restores p53 function (P53R3) were tested on the SKPC-1, MIA PaCa-2, and BxPC-3 human pancreatic cancer cell lines and compared their effects with the conventional antitumoral drug gemcitabine. The drugs were used at variable concentration ranging from 0.5 to 50 µM and cell viability for each cell line was assessed by the colorimetric MTS assay on 2D. As it is showed in Figure 1, all drugs decreased cell viability at 48 h in a dose-dependent manner, compared with untreated cells. Some differences were found between cell lines, with the BxPC-3 cell line being the most sensitive to the compounds tested.

Analyzing the half-maximal inhibitory concentration (IC_50_) (Supplementary Table S1), the triple epigenetic inhibitor UVI5008 showed the highest effect in all pancreatic cell lines tested, similar to previous reports on leukemia, colon, prostate, and breast tumor models, with a good safety profile in mice [28]. The reversion of the epigenetic modifications performed by methyltransferases and deacetylases induces apoptosis on tumoral cells, mediated by activation of the death receptor pathway, production of reactive oxygen species (ROS), and reactivation of tumor suppressor genes such as p21 [28].

BIX01294 was the second most potent drug, inducing a dose-dependent effect on all pancreatic lines analyzed, especially on the MIA PaCa-2 cells. This drug selectively impairs the G9a HMTase and also shows inhibitory activity towards the nuclear receptor binding SET Domain Protein 1 (NSD1), a bifunctional transcriptional regulatory protein that encodes a histone methyltransferase that is mutated in several tumors, including pancreatic cancer [29]. Its antitumoral effect was also described in nasopharyngeal carcinoma, ovarian cancer, or cholangiocarcinoma, either alone or in combination with other therapies [30,31,32].

MS275 showed a similar cell inhibition (IC_50_ around 14–18 µM) on all cell lines, even to the partial gemcitabine-resistant MIA PaCa-2 cells. This HDAC1/4 inhibitor showed a significant improvement in overall survival in combination with an anti-PD1 therapy in a murine bladder cancer model [33] and was tested in clinical trials for resistance to breast cancer in combination with other therapies like anti-PD-1 [34] or the estrogen inhibitor exemestane [35]. Recently, the potential of this drug to revert the immunosuppressive tumor environment in combination with an immunostimulant therapy (IL-12) showed very promising results in murine colon carcinoma and breast tumor models with a poor response to anti-PDL1 therapies [36]. The molecular mechanism of the MS275 antitumor activity relies on the activation of the TNFSF10 promoter and the expression of TRAIL (Apo2L, TNFSF10), which triggers tumor-selective apoptosis. In addition, MS275 also induces cell cycle arrest in acute myeloid leukemia cells mediated by the expression of p21 and independently from TRAIL [37].

In the case of P53R3, inhibition of the cell growth activity was similar on the three cell lines (IC50:12–15 µM). It is important to note that MIA PaCa-2 cells have one of the mutations restored by P53R3 (p53R248W) [27], being different from those detected in BxPC-3 (p53Y220C) and SKPC-1 cells (p53R248L) [38]. For that reason, P53R3 could be a good adjuvant therapy in PDAC patients carrying p53 mutations, apart from the ones targeted directly by the molecule.

With the other drugs, MIA PaCa-2 and BxPC-3 cells were quite resistant to 1 (IC_50_ over 50 µM), and psammaplin A, an analogue of UVI5008, showed higher levels of IC_50_ in the three cell lines (Supplementary Table S1).

Regarding gemcitabine, the deoxycytidine nucleoside analogue, it induced cell death on the BXPC-3 cell line at low concentration (IC_50_: 2 µM), followed by the SKPC-1 cells (IC_50_: 21 µM). The MIA PaCa-2 cell line, as expected, was the most resistant one (IC_50_ >50 µM) [39].

Apart from *p53*, the three cell lines differ in other genetic mutations that could be responsible of the different efficacy found with the drugs tested. For instance, while SKPC-1 and MIA PaCa-2 carry a mutation in the *KRAS* gene (G12D and G12C, respectively) and a homozygous deletion of *CDKN2A* (encoding p16), the BXPC-3 cell line has a wild-type genotype for both genes. *DPC4/SMAD4* is absent in SKPC-1 and BXPC-3 cells but it is included on the MIA PaCa-2 cells [40,41,42].

In summary, all the epigenetic drug inhibitors tested were able to decrease cell viability, measured by the MTS assay at 48 h on 2D, but with different effects depending on the target cell lines and dose. Compounds UVI5008 (HDAC1/4, SIRT1, and DNMT3a inhibitor) and BIX01294 (EHMT2 inhibitor) were the most promising ones, followed by MS275 (HDAC1/4 inhibitor) and psammaplin A (HDAC1 and DNMT1 inhibitor).

### 2.2. Kinetic Effect of the Anti-Tumoral Drugs in the Pancreatic Cell Lines

Many cell viability studies traditionally perform a single technique that usually relies on the use of end-point tests in two-dimensional (2D) cell cultures. However, there are several methods that can provide very useful complementary information, such as real-time tests and three-dimensional (3D) cell cultures, which are increasingly used in cancer research not only for the characterization of drug efficacy, but also to study different events implicated in the tumorigenesis, proliferation, or the tumor environment [43].

To study in detail the inhibitory effect induced by the anti-tumoral drugs on the adherent pancreatic cell lines, we also used the xCelligence RTCA system. The method measures the cellular impedance, and permits us to monitor, in real-time, the viability and proliferation of cells. We treated SKPC-1, MIA PaCa-2, and BxPC-3 cells with all the compounds and gemcitabine at different concentrations, considering the IC_50_ concentrations obtained from the MTS assay.

**Figure 2:**
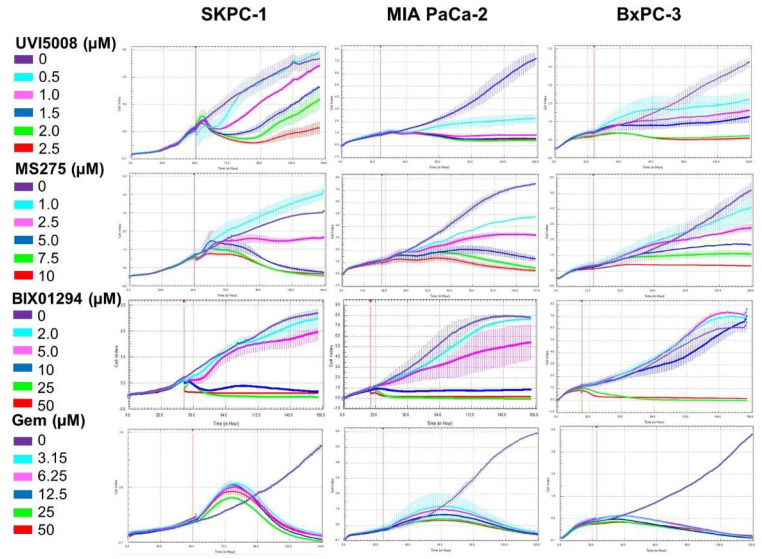
Kinetics of the cell viability of SKPC-1, MIA PaCa-2, and BxPC-3 cells incubated with the epigenetic inhibitors UVI5008, MS275, and BIX01294 or gemcitabine (Gem). The treatments were added after cell stabilization (vertical red lines). Cell viability was monitored with an impedance-based real-time cell analyzer.

In Figure 2, the real-time behavior of the three PDAC cell lines in the presence of UVI5008, MS275, BIX01294, and gemcitabine is shown (the other compounds are represented in Supplementary Figure S1). As expected, the three epigenetic inhibitors decreased cell viability on all cell lines tested, although timing was very important. For example, MS275 was not very effective at 24 h, but induced inhibition at 48 h and further on. On the contrary, UVI5008 and BIX01294 affected viabilities at even earlier times. Full analysis of IC_50_ can be found in Supplementary Table S2. Again, psammaplin A was the next most effective compound, requiring 1 and P53R3 a higher concentrations to reach the IC_50_.

It is worthy of note that thanks to this technique, it was possible to see that gemcitabine induces proliferation of the cells at early stages (especially to the SKPC-1 cells) (Figure 2), followed by a decrease on the cell viability mostly at 72 and 96 h. Similarly, to what it was found by the MTS assay, we did not see a dose-dependent effect being very efficacious at very low concentrations. Interestingly, there was a relevant decrease in the cell index (CI) after 48 h at low concentration in MIA PaCa-2 cells, as seen by the RTCA, although the cell metabolism was not significantly affected as seen in the MTS assay (Figure 1 and Supplementary Table S1). These differences between the two cell viability assays have also been described for other compounds and are presumably due to the different events monitored in each assay [44,45]. While the MTS assay measures cells that are metabolically active, RTCA analyses the changes in the impedance upon attachment or detachment of cells to the gold electrodes at the bottom of the wells. Therefore, changes in the adherence of the MIA PaCa-2 cells in the presence of gemcitabine precede to cell death.

In summary, the analysis of cell proliferation at real time provided important information about cell behavior with the different treatments, especially at early time points. The most potent inhibitors for all type of cells were again UVI5008, MS275, and BIX01294, followed by psammaplin A.

### 2.3. Effect of the Anti-Tumoral Drugs on 3D Cultures

The data obtained using the colorimetric MTS and the real-time system xCelligence assays, although representative of the inhibitory effect of the drugs on PDAC cells, are both methods based on cell culture in 2D. For this reason, we introduced the three-dimensional (3D) cell culture, which better mimics the tumor architecture.

**Figure 3:**
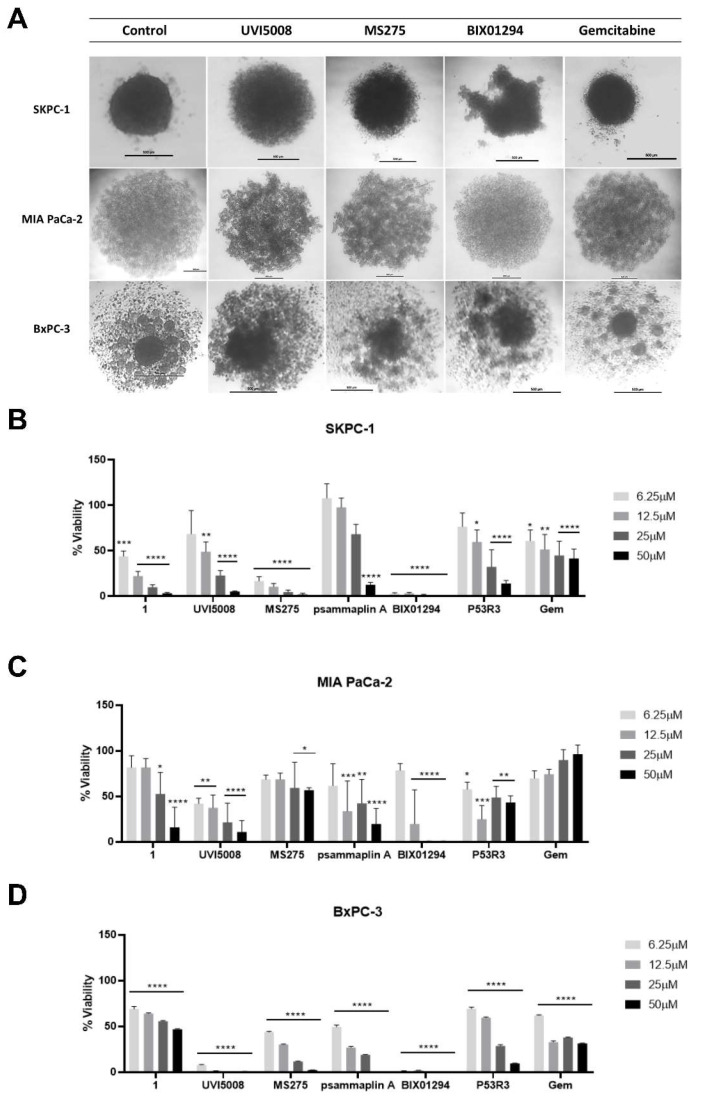
Antitumoral effect induced by the epigenetic inhibitors 1 (MS275 analogue), 2 (UVI5008), 3 (MS275), 4 (psammaplin A), 5 (BIX01294), and 6 (P53R3) and gemcitabine (Gem) in SKPC-1, MIA PaCa-2, and BxPC-3 spheroids. (**A**) Optical microscope images of the spheroids: untreated (control), and treated with UVI5008, MS275, and BIX01294 at the highest concentration tested (50 µM). Scale: 500 µm. Percentage of cell viability on the SKPC-1 (**B**), MIA PaCa-2 (**C**), and BxPC-3 (**D**) spheroids treated with the anti-tumoral drugs at four different concentrations. Statistically significant differences compared to untreated cells were represented as: * *p* ≤ 0.05, ** *p* ≤ 0.01, *** *p* ≤ 0.001, **** *p* ≤ 0.0001.

In the case of SKPC-1 cells, they form a compact spheroid structure, with defined edges and a considered viable size (400–500 µm) (Figure 3). On the contrary, BxPC-3 and MIA PaCa-2 spheroids were less compact. In MIA PaCa-2, the spheroids had a diameter bigger than 1 mm, even using methylcellulose at 0.24%, which was described to help in the spheroid formation [46]. Although this could represent a problem, because large spheroids (>500 µm) could contain a hypoxic or necrotic core [47], MIA PaCa-2 spheroids resulted to have viable and functionally active cells (data not shown). Once established the spheroids conditions, they were treated with variable concentrations (6.25 to 50 µM) of the compounds and gemcitabine as control for 72 h. We tested the viability based on quantitation of the ATP, which is a marker of metabolically active cells.

The spheroids treated with some of the compounds had a significant change in their shapes (Figure 3A). Both, SKPC-1 and BxPC-3 spheroids showed a dispersed form, losing compactness in the external area, while MIA PaCa-2 cells modified the three-dimensional structure, forming cracks in the spheroid. The viability assay performed on those cells growing in 3D revealed that the epigenetic inhibitors were even more effective than gemcitabine.

Again UVI5008, MS275, and BIX01294 showed a higher inhibitory effect on SKPC-1 and BxPC-3 cells (Figure 3B–D); 1, psammaplin A. and P53R3 required higher doses to reach IC_50_ (Supplementary Table S3), except for 1 on SKPC-1 cells (IC_50_ 5 µM). Gemcitabine showed preferential effect on BxPC-3 cells (IC_50_ 8 µM), being MIA PaCa-2 resistant to gemcitabine, even at the highest dose tested (50 µM). The MIA PaCa-2 spheroids showed a particular resistance not only to gemcitabine, but also to the epigenetic inhibitors 1 and MS275 (Figure 3 and Supplementary Table S3). Because methylcellulose was required to form the spheroid on this type of cell line, we cannot exclude potential interferences with the drug effects. This will require further studies to confirm this assumption.

The relevance of the extracellular cell matrix (ECM) in 3D cell cultures to characterize the antitumor effect of epigenetic inhibitors was already described for the Enhancer of Zeste 2 (EZH2) histone methyltransferase in ovarian cancer cells [48]. While in 2D cell culture the EZH2 inhibitor showed a limited efficacy, in 3D cultures it was able to decrease cell growth and invasion, and to induce apoptosis pointing to a relevant role of the ECM in the cell sensitivity to the compound. Similarly, P53R3 shows a two- to threefold increased efficacy in all the spheroids, compared to the monolayer culture conditions. The reason for this increased activity could be due to two main signaling mechanisms that trigger p53 activation: (i) the anchorage-independent activation of mutated KRAS, which is inhibited in monolayer cultures [49] and (ii) the cellular stress induced by the hypoxic core of the spheroids, which is also present in many tumors [50].

Our results analyzing the viability assay using the 3D growth of cells, which can simulate better the in vivo situation, shows that our epigenetic inhibitors are more potent than gemcitabine, opening the possibility of using those drugs for gemcitabine-resistant tumors, either alone or in combination.

### 2.4. Synergistic Effect with Gemcitabine

**Figure 4:**
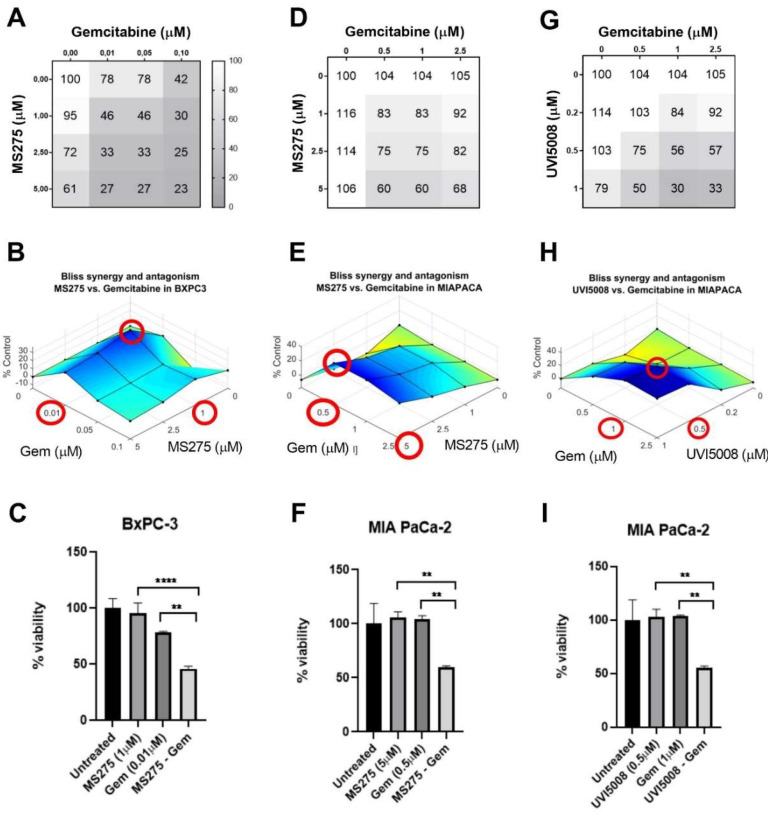
Synergistic antitumoral effect induced by gemcitabine in combination with MS275 in BxPC−3 (**A**–**C**) and MIA PaCa−2 cells (**D**–**F**), or with UVI5008 in MIA PaCa−2 cells (**G**–**I**). Cell viability matrix (**A**,**D**,**G**); (bliss synergy graphs **B**,**E**,**H**). Red circles: highest synergy score and the associated concentrations; (**C**,**F**,**I**). Cell viability of BxPC−3 and MIA PaCa−2 cells with the combinations and the individual drugs at the concentrations that showed the highest synergistic effect. Statistically significant differences compared to untreated cells were represented as: ** *p* ≤ 0.01 and **** *p* ≤ 0.0001.

In many tumors, combinations of chemotherapies have demonstrated to be useful, showing in some cases synergistic effect, allowing for the reduction of individual drug doses and the potential secondary harmful effects. To investigate the effect of combining our compounds with gemcitabine, we treated MIA PaCa-2, SKPC-1, and BxPC-3 cells, with variable doses of drugs in combination with gemcitabine for 48 h and analyzed cell viability. Once obtained the percentage of cell viability (Figure 4A,D,G shows some examples with MS275 and UVI5008), we calculated the synergistic score to discriminate if a combination between two drugs gives antagonistic, additive, or synergic effects (see Materials and Methods).

From the different concentrations tested (Supplementary Table S4), we found two drugs inducing synergy to gemcitabine, UVI5008, and MS275. The highest synergy score was found for the combinations: (i) 1 µM MS275 plus 0.01 µM gemcitabine on BxPC-3 cells (Figure 4B); (ii) 5 µM MS275 plus 0.5 µM gemcitabine on MIA PaCa-2 cells (Figure 4E); (iii) 0.5 µM UVI5008 plus 1 µM of gemcitabine on MIA PaCa-2 (Figure 4H).

We found that the inhibition of cell proliferation, using the combination of both drugs at very low concentration, was much higher (statistically significant) than those obtained with the individual drugs, either MS275 in two cell lines (Figure 4C,F) or UVI5008 in the MIA PaCa-2 cells (Figure 4I).

Our results corroborate those from Ma and coworkers [19], who found a synergistic effect of Entinostat (MS275) and gemcitabine in different human pancreatic cell lines with diverse sensitivity to gemcitabine. Likewise, Ivaltinostat (CG-200745), another HDAC inhibitor that targets histone H3, which showed good anti-proliferative activity in the BxPC3, Cfpac-1, and HPAC cells lines (IC_50_; 2.4, 10.7 and 7.4 μM, respectively), also showed synergistic effect with gemcitabine/erlotinib using an IC_20–30_ of each drug [51].

The use of the triple epigenetic inhibitor UVI5008 in combination with gemcitabine showed an efficient anti-proliferative activity in MIA PaCa-2 cells, similarly to what is found with MS275. Thus, both compounds could be a good alternative on a personalized treatment for PDAC, especially in gemcitabine-resistant tumors.

The other epigenetic inhibitors tested in this study (1, psammaplin A, BIX01294) and the molecule that restores the p53 function (P53R3) only showed additive effects to gemcitabine. Moreover, none of the compounds used showed synergy on the SKPC-1 cell line (data not shown).

### 2.5. Hemocompatibility of the Epigenetic Inhibitors

To discard any potential toxicological effect on non-tumoral cells, the hemocompatibility of the epigenetic inhibitors that showed the highest antiproliferative effect in the PDAC cell lines (UVI5008, MS275, and BIX01294), gemcitabine, and the synergistic combinations was characterized in whole blood samples and peripheral blood mononuclear cells (PBMCs) from healthy donors as described in the Supplementary Information. We performed a hemolysis test with the blood samples and characterized the potential toxic effect on PBMCs by flow cytometry, labelling the cells with Annexin V/iodide propidium to detect cell death by apoptosis or necrosis. None of the compounds or their combinations induced significant hemolysis (Supplementary Figure S2A) or cell toxicity on human PBMCs (viability ≥85%) (Supplementary Figure S2B), indicating the biocompatibility of these compounds on healthy cells.

### 2.6. Transcriptome Analysis Shows Differences in Gene Expression among Treatments and Cell Lines

**Figure 5:**
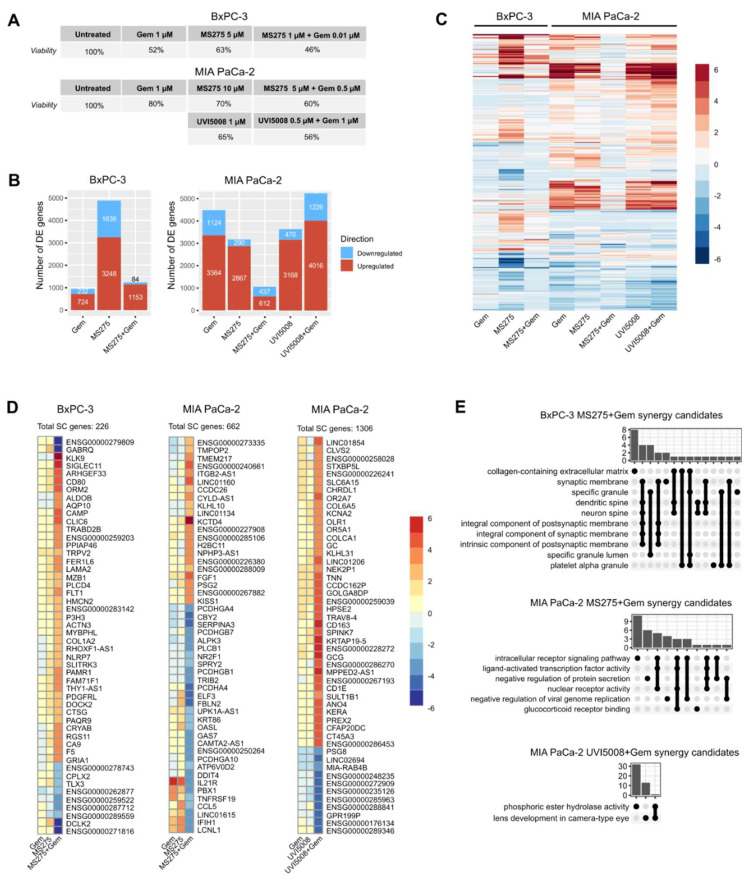
Transcriptome analysis of the synergistic effect of gemcitabine (Gem) with MS275 on BxPC−3 and MIA PaCa−2 cells, and with UVI5008 on MIA PaCa−2 cells. Drug combinations and doses selected for RNA-Seq analysis and percentages of cell viability (**A**). Number of upregulated (red) and downregulated (blue) genes induced by treatments when compared to untreated control (**B**). Heatmap representation of all differentially expressed genes induced by treatments when compared to untreated control (**C**). Top 50 of synergy candidate (SC) genes ordered by fold change in each treatment combination (**D**). Genes are colored according to their expression levels. Top 10 over-representation analysis of synergy candidate genes using Gene Ontology (**E**). Bars represent the number of genes in the intersection among terms.

To comprehend the basis behind the synergy for UVI5008 or MS275 with gemcitabine, we performed RNA-Sequencing on BxPC-3 cells (treated with MS275+Gem), and MIA PaCa-2 (treated with either UVI5008+Gem or MS275+Gem) and compared the results with those found for individual treatments. We selected those doses inducing the highest synergistic score for the combinations and similar cell viability with the individual drugs (Figure 5A). Cluster analysis of transcripts per million (TPM) displayed markedly different transcriptional profiles among samples, and accurately classified most of them based on both cell lines (BxPC-3 or MIA PaCa-2) and treatment (Supplementary Figure S3).

Figure 5B summarizes the number of differentially expressed (DE) genes, most of them upregulated, in both cell lines with the individual and combined treatments. The complete lists of DE genes can be found in Supplementary Table S5.

We asked whether those DE genes were common among treatments (Supplementary Figure S3B). In BxPC-3, more than three hundred genes were shared among the three treatments, although there were some unique DE genes for each therapy. Similar observations were found in MIA PaCa-2 cells. Despite the reduced number of DE genes in these cells using the combination MS275+Gem, a good proportion of genes were uniquely DE (555 genes) compared to the respective single treatments. UVI5008+Gem shared more than 3000 DE genes with either gemcitabine or UVI5008 alone, but few DE genes where shared among the individual treatments (183).

A clear picture can be obtained by depicting the expression level of all DE genes among all groups (Figure 5C). MS275 generates a unique transcriptional signature in BxPC-3, while treatment with gemcitabine or MS275+Gem displayed a similar profile (it is important to note that gemcitabine dose was 100 times reduced in the MS275+Gem combination). In MIA PaCa-2 cells, either gemcitabine, MS275, UVI5008, or UVI5008+Gem shares a similar signature, while the combined MS275+Gem therapy outstands with fewer expression changes and, worthy to note, a much higher proportion of downregulated genes.

### 2.7. Gene Set Enrichment Analysis (GSEA) Differentiates Molecular Patterns among Treatments

We performed (GSEA) to find cellular mechanisms triggered by the treatments (Supplementary Table S6). In BxPC-3 cells, gemcitabine induced downregulation of fatty acid metabolism and DNA replication (Supplementary Figure S4A). MS275 negatively correlated with multiple pathways, including Myc targets, mTOR signaling, DNA replication, and interferon response, but induced upregulation of Hedgehog signaling, related with cancer progression. MS275+Gem displayed similar signatures than MS275 alone, but it was also enriched in routes associated to pro-tumorigenic events like upregulation of KRAS signaling and epithelial–mesenchymal transition.

In MIA PaCa-2, most transcriptomes matched with similar molecular signatures (Supplementary Figure S4A), and as it was found on BxPC-3, some of them were related with pro-tumorigenic events. A distinct signature was found in the combination MS275+Gem, with two items suggesting the downregulation of estrogen response. When inspecting the core enrichment genes implicated in the estrogen-related signatures, we obtained a total of 74 downregulated genes in MIA PaCa-2 cells when the combined MS275+Gem therapy was used (Supplementary Figure S4B).

### 2.8. Identification of Unique DE Genes in Combinatory Treatments

To characterize the genes uniquely mobilized by the synergy combination, we filtered the DE genes in the drugs combinations, keeping those that were not DE by either gemcitabine or the corresponding epigenetic inhibitor (UVI5008 or MS275), and included those common DE genes with contrary fold change direction in gemcitabine or the inhibitor.

We obtained a total of 226 synergy candidate (SC) genes in BxPC-3 cells treated with MS275+Gem, and 662 and 1306 in MIA PaCa-2 cells treated with MS275+Gem and UVI5008+Gem, respectively (Supplementary Table S7). The top 50 genes with highest absolute fold changes are depicted in Figure 5D. The biotypes of the SC genes are represented in Supplementary Figure S5A.

In BxPC-3 cells treated with MS275+Gem, the most downregulated gene was *ENSG00000279809*, a novel transcript of unknown function, followed by *GABRQ*, codifying for Gamma-Aminobutyric Acid (GABA) Type A Receptor Subunit Theta. GABA has been related with pancreatic cancer growth [52], and *GABRQ* was reported to mediate proliferation in hepatocellular carcinoma [53]. Therefore, its marked downregulation could be actively affecting the viability of the cells. Another major downregulated SC gene, Doublecortin-like kinase 2 (*DCLK2*), is a microtubule-associated protein with polymerizing and kinase activity. Although no major roles in carcinogenesis were described for this gene, its family member *DCLK1* is upregulated in a variety of cancers including PDAC, being a potential therapeutic target [54].

The most upregulated genes included *KLK9, SIGLEC11, CAMP, ORM2, CD80, ARHGEF33,* and *CLIC6*. Kallikrein-related Peptidase 9 *(KLK9)*, reported as a favorable prognostic marker in ovarian cancer [55], was highly upregulated by the combination of drugs, as was Orosomucoid 2 (*ORM2*), a tumor suppressor in liver cancer [56]. Nonetheless, the other upregulated genes have been associated to pro-tumorigenic roles and poor prognosis: *SIGLEC-11* [57], *CAMP* [58], *CD80* [59], and *CLIC6* [60].

To explore the biological implications of those SC genes on the BxPC-3 cells treated with MS275+Gem, we also performed Gene Ontology (GO) over-representation test (Figure 5E, Supplementary Table S8). Two main clusters appeared, one related to components of the “synaptic membrane”, and the other to “secretory granule lumen and collagen-containing extracellular matrix”. In the “synaptic membrane”-related GO clusters, we found diverse upregulated genes mostly related to pro-tumorigenic roles (as *GRIA1* and *CHRNB1, SLITRK3, CRYAB,* or *ADORA1*), but also to the aforementioned *GABRQ*. The pathway “secretory granule lumen” included upregulated *AOC1, CAMP, CTSG, DOCK2, F5, LYZ, ORM2, PRTN3,* and *VWF*. The “collagen-containing extracellular matrix” pathway was enriched by upregulated *LAMA2*, *CTSG* (cathepsin G), various collagen chains, and *ORM2*, and might indicate alterations in cell–cell adhesion mechanisms.

In the MIA PaCa-2 cell line, the MS275+Gem combination generated a different signature. The most upregulated gene was *KCTD4* (codifying for potassium channel tetramerization domain containing 4), followed by *FGF1* (fibroblast growth factor 1). Although there are no described functions for *KCTD4* in PDAC, the family of KCTD proteins have been recently described as regulator proteins in cancer, with either tumor-suppressive or tumor-stimulation functions, and as potential therapeutic targets [61]. *FGF1* is implicated in the promotion of the Myc signaling pathway [62], a route upregulated in pancreatic cancer that was inhibited by MS275. 

The upregulation of pro-tumorigenic or pro-survival responses observed may be a cause of the direct action of the drugs, or reflecting compensatory escape pathways [63,64,65].

In fact, compensatory mechanisms have been described for treatments targeting the Kras effectors MEK1/2, Erk1/2 or Akt also in pancreatic cancer (either used individually or in combination), or also with antihormone therapy in breast cancer cells [66,67].

For that reason, the use of combination of drugs with different antitumor mechanisms can be the key to overcome the compensatory escape pathways involved in drug resistance. In fact, combining nanoliposomal irinotecan and benzoporphyrin derivative-based photodynamic therapy (PDT) has showed about threefold higher tumor growth inhibition, compared to the individual treatments [68]. PDT is able to reduce the expression of the ATP-binding cassette G2 (ABCG2) transporter, induced by irinotecan, which mediates the efflux of the drug from the cells. In agreement with our results, the combination induced a therapeutic effect lower doses than the individual treatments.

Among the most downregulated SC genes, we found interesting genes related to cancer growth: *ELF3*, a transcription factors that could promote the progression of pancreatic cancer [69]; *SERPINA3*, reported to increase tumor invasion and a correlate of poor prognosis in several types of cancer [70,71]; and Fibulin-2 (*FBLN2*), a glycoprotein part of the extracellular matrix [72]. Although the interleukin-21 receptor is mostly known for its role in immune signaling, *IL21R* was detected in various cancer types, and its suppression could hamper cell proliferation and invasion [73].

Diverse protocadherin genes were also downregulated (*PCDH9, PCDHA4, PCDHGA10, PCDHGA4, PCDHGB1, PCDHGB7, and PCDHGC3)*, as well as P-cadherin (*CDH3*). Cadherin molecules are known to be implicated in cancer progression, and P-cadherin is abnormally upregulated in PDAC and related to poor prognosis [74]. Their downregulation might be impairing MIA PaCa-2 proliferation, as well as the DNA inhibitors *ID1, ID2,* and *ID3* [75]. Furthermore, we found 17 downregulated candidates that were also contributing to the estrogen signature found in by GSEA, i.e., *ELF3*, *SERPINA3*, *SGK1*, *SCNN1A*, *DLC1*, *KRT15*, *KRT19,* or *THSD4*. Estrogen signaling play a major role favoring proliferation in some cancers [76]. The inhibition of this signaling pathway by MS275+Gem could be responsible for the antiproliferative effect observed on MIA PaCa-2 cells.

The GO over-representation analysis of the SC genes on MIA PaCa-2 cells treated with MS275+Gem did not indicate new altered pathways, but it reflected some downstream changes in transcription (Figure 5E). They included “intracellular receptor signaling pathway”, “ligand-activated transcription factor activity”, and “nuclear receptor activity” pathways. Those pathways shared similar nuclear receptor genes as *NR4A3* (upregulated), *NR2F1, NR3C2, NR4A2,* or *NR4A1* (downregulated), and hormone nuclear receptors as *ESR*2 (upregulated), *SREBF1, THRA,* or *RORC* (downregulated). The most enriched route, “intracellular receptor signaling pathway”, was mostly composed by downregulated genes including *AKR1C3, CYP26B1, IFIH1, NR2F1, OASL,* transcription factors (*TP63*, *RORC*), regulatory elements (*SREBF1, SFRP1*), diverse nuclear receptors (*NR2F1, NR3C2, NR4A1, NR4A2, THRA),* and the zinc-finger proteins *ZMIZ1* and *ZNF536.*

The combination UVI5008+Gem in MIA PaCa-2 cells was characterized by a high number of SC genes. When inspecting all the candidate list, we found both pro and anti-tumorigenic signatures. The most upregulated genes included *ENSG00000228272* (long non-coding RNA), *KRTAP19-5*, *MPPED2-AS1*, *CD163, GCG*, or *SPINK7*, whereas the top downregulated genes included diverse novel protein-coding and various long non-coding RNAs.

Other SC genes in the list with expected anti-tumor effect were upregulated, such as *HPSE2* (heparanase 2) [77]. Similarly, genes implicated in tumorigenesis or progression were downregulated by the combinatory treatment, like *SPRY1* (sprouty RTK signaling antagonist 1) [78], *CDK15* (Cyclin-dependent kinase 15) [79], or *VIM* (vimentin) [80]. The checkpoint inhibitor *CTLA4* (Cytotoxic T-Lymphocyte Antigen 4) was downregulated as well, which could potentiate the immune response in vivo when using the combination of UVI5008+Gem [81].

MIA PaCa-2 cells treated with UVI5008+Gem only reported two enriched pathways: “lens development in camera-type eye” and “phosphoric ester hydrolase activity”, with no obvious role in carcinogenesis (Figure 5E).

### 2.9. Shared SC Genes between Treatments

Lastly, we checked whether SC genes were shared among the drug combinations in both cells lines (Supplementary Figure S5B,C) that could correlate with a common antitumoral effect.

MS275+Gem shared six candidate genes in BxPC-3 and MIA PaCa-2: *ENSG00000289559, ITGB1-DT, KRT80, PCDHGA10, SMPDL3B,* and *ZNF385C*.

MS275+Gem and UVI5008+Gem shared 10 candidates in BxPC-3 cells and MIA PaCa-2 cells, respectively: *ABCC9, BOC, ENSG00000285804, TFEC, NBPF4, CTSG* (upregulated), *DCLK2, ENSG00000259522 ENSG00000262877,* and *PCDHGA10* (downregulated). In MIA PaCa-2 cells, MS275+Gem and UVI5008+Gem shared more genes, 86 candidates. However, among all the combinations, the downregulation of *PCDHGA10* was the only DE gene in common. Oddly, this gene is already downregulated in pancreatic cancer cells by methylation of its promoter by DNMT3b [82,83], which is not a target of UVI5008 or MS275.

### 2.10. Kaplan–Meier Estimate of SC Genes’ Expression in PDAC Patients

To highlight those SC genes with described clinical relevance, we utilized the Kaplan–Meier online tool to correlate gene expression with survival in a subset of PDAC patients. We plotted all SC genes whose upregulated or downregulated expression was significantly related with survival (Supplementary Figures S6–S12). Many SC genes were significantly related to an increased survival, such as the case of *GRIA1*, upregulated on BxPC-3 cells treated with MS275+Gem, *CDH3, TP63, KRT19, SCNN1A,* and *OASL*, downregulated on MIA PaCa-2 cells treated with MS275+Gem.

The molecular changes described in this section were found exclusively in the synergy combination of gemcitabine with the epigenetic inhibitors MS275 or UVI5008. The described mechanisms might be contributing to the synergistic impairment of tumor growth induced by the drug combinations. Although much work remains to comprehend the basis beneath drug synergy, some of these findings could help predicting PDAC response to combined chemotherapy.

Besides, future in vivo testing in a relevant PDAC mouse model and in patient-derived organoids is needed to try to translate the results observed in the present study to the clinic. In addition, finding an appropriate nanotransporter for the hydrophobic epigenetic inhibitors could also increase the efficacy in vivo and reduce the therapeutic dose and the potential systemic toxicity.

## 3. Materials and Methods

### 3.1. Cell Culture

Human pancreatic cell lines SKPC-1, MIA PaCa-2, and BxPC-3 were provided by Carmen Guerra at Spanish National Center for Cancer Research (CNIO), Spain.

SKPC-1 and MIA PaCa-2 were cultured in Dulbecco’s Modified Eagle Medium (DMEM, Corning, Thermo Scientific, Madrid, Spain), supplemented with 10% fetal bovine serum (FBS, Merck, Darmstadt, Germany), 100 U/mL Penicillin, 100 μg/mL Streptomycin, and 1 mM Sodium Piruvate (Gibco, ThermoFisher, Madrid, Spain) at 37 °C and 5% CO_2_. BxPC-3 was cultured in Roswell Park Memorial Institute medium (RPMI 1640 medium, Corning, New York, USA) supplemented with 10% FBS, 100 U/mL Penicillin, and 100 μg/mL Streptomycin at 37 °C and 5% CO_2_. Cells were subcultured every 2–3 days (2–10 passages).

### 3.2. Anti-Tumoral Drugs

**Scheme 1:**
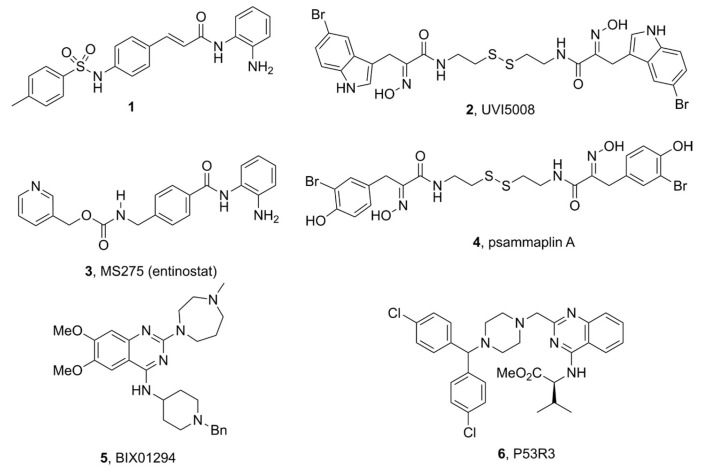
Chemical structure of 1 (MS275 analogue), UVI5008, MS275, psammaplin A, BIX01294, and P53R3.

Six different compounds were synthesized by the Organic Chemistry Innovative Designs (ORCHID) group. Five of them correspond to epigenetic inhibitors: 1 [16], UVI5008 [17,18], MS275 [35], psammaplin A [21,22], and BIX01294 [24,25]. Besides, P53R3, a molecule that restores the p53 wt function [27], was also tested. Scheme 1 shows the chemical structure of each drug. All compounds were >95% pure by HPLC/MS analysis.

Gemcitabine was purchased from Carbosynth (Berkshire, UK). Compound stocks were dissolved in dimethyl sulfoxide (DMSO 1%) prior to their use. DMSO was provided by Sigma-Aldrich (Merck, Darmstadt, Germany).

### 3.3. Cell Viability Assays

#### 3.3.1. MTS Cell Proliferation Assay

Cells were seeded in 96-well plates at densities of 7 × 10^3^ cells/well (MIA PaCa-2 and SKPC-1) and 6 × 10^3^ cells/well (BxPC-3), incubated for 24 h (MIA PaCa-2 and BxPC-3) or 48 h (SKPC-1) to allow for their adherence to the plate surface prior to the addition of the treatments. The cells were subsequently treated with different doses of the drugs. DMSO concentration in media never reached above 0.1%. After 48 h of treatment, a colorimetric cell viability assay was performed using the Cell Titer 96^®^ AQueous One Solution Cell Proliferation Assay kit (MTS, Promega, Wisconsin, WI, USA) according to the manufacturer’s instructions. Media alone and media with the drugs were used as background controls and untreated cells as negative control, respectively. Plates were further incubated for 2 h with the MTS reagent and supernatants measured at 490 nm on an Envision multidetector (Perkin-Elmer Inc, Connecticut, CT, USA). Cell viability, expressed as a percentage, was calculated as described in Equation (1):

% viability = ([A]treatment/[A]control) × 100,(1)

where [A]treatment is the absorbance of the cells incubated with the drugs minus the absorbance of the drugs, and [A]control is the absorbance of the untreated cells minus the absorbance of the culture medium.

#### 3.3.2. xCELLigence Real-Time Cell Analyzer (RTCA)

Cells were seeded into 96-well E-Plates 16 (ACEA, Biosciences, Agilent, California, CA, USA) at densities of 7 × 10^3^ (MIA PaCa-2), 10^4^ × 10^3^ (SKPC-1), and 6 × 10^3^ cells/well (BxPC-3) and rested for 24 h (MIA PaCa-2 and BxPC-3) or 48 h for (SKPC-1) to allow for the cells to adhere. Later, drugs were added at different concentrations. Cell growth was dynamically monitored with the xCELLigence RTCA (Agilent Technologies, California, CA, USA) system for 96 h. Cell viability at different time points, expressed as a percentage, was calculated as described for the MTS colorimetric assay by using the cell index (impedance) values instead of absorbance.

#### 3.3.3. Spheroid Formation and Cell Viability in 3D Cultures

After optimization of the number of cells, 3 × 10^3^ SKPC-1 cells, 10^4^ MIA PaCa-2 cells, and 10^4^ BxPC-3 cells were cultured in hanging drops using Perfecta3D™ Hanging Drop Plates (3D Biomatrix, Michigan, MI, USA) in 40 μL of culture medium. To improve the formation of the spheroid, MIA PaCa-2 cells were supplemented with 0.24% methyl cellulose (M0512, Sigma-Aldrich, Merck). Spheroidal colonies grew on the bottom of the wells at 37 °C in atmosphere containing 5% CO_2_, for 4 days, when the spheroid diameter reached 500–600 µm, except MIA PaCa-2 spheroids, that reached more than 1 mm of diameter. Once the spheroids formed, they were incubated with the compounds at different concentrations for 72 h. Afterwards, they were plated in a 96-well black bottom-clear plates (BD Falcon, Fisher Scientific, Madrid, Spain) and cell viability was measured using the CellTiter-Glo^®^ 3D Assay reagent (Promega, Milan, Italy). Luminescence was read after 30 min with an Envision multidetector (Perkin-Elmer Inc Norwalk, Connecticut, CT, USA). Cell viability, expressed as a percentage, was calculated as described for the MTS colorimetric assay by using the luminescence values instead of the absorbance. Images of the spheroids were taken using the inverted microscope Nikon ECLIPSE Ti (Nikon Instruments Inc., Tokio, Japan).

#### 3.3.4. Synergy Analysis

Cell viability studies with combinations of the epigenetic inhibitors and gemcitabine at different concentrations were performed as described for the MTS proliferation assay.

The extent and direction of drug combination effect in PDAC viability were determined by Combenefit software [84], based on the Bliss Independence model. The Bliss Independence model quantify the degree of synergy where the expected response is the multiplicative effect of single drugs as if they act independently [85,86]. According to the software developers, a score below −25 indicates an antagonistic effect, between −25 and 25 means an additive effect, and > 25 means synergistic effect.

### 3.4. Statistical Analysis

The 50% inhibitory concentration (IC_50_) values were defined as the drug concentrations required to reduce cellular viability to 50% of the untreated control cells. Graphpad Prism version 8 for Windows (GraphPad Software; California, CA, USA) was used for statistical analysis and graph representation. A Shapiro–Wilk test was conducted to determine the distribution of the samples, followed by an Anova or Kruskal–Wallis test to ascertain significant differences between control and treatments. All data are expressed as mean ± standard deviation (SD) from three independent measurements. Statistically significant results are referred to as: * *p* ≤ 0.05, ** *p* ≤ 0.01, *** *p* ≤ 0.001, **** *p* ≤ 0.0001.

### 3.5. RNA Sequencing

For the RNA-Seq experiment, cells were cultured in 2D following the conditions de-scribed for the synergy experiments.

BxPC-3 cells were treated with either 1 µM gemcitabine (Gem), MS275 (5 µM), MS275 (1 µM) + Gem (0.01 µM), or left untreated for 48 h. MIA PaCa-2 cells were treated with either Gem (1 µM), MS275 (10 µM), UVI5008 (1 µM), MS275 (5 µM) + Gem (0.5 µM), UVI5008 (0.5 µM) + Gem (1 µM), or left untreated for 48 h. Cells were harvested and RNA was extracted using PureLink™ RNA Mini Kit (Invitrogen, Massachusetts, MA, USA), according to the manufacturer’s instructions. RNA quality was assessed using Agilent 2100 Bioanalyzer and the Agilent RNA 600 Nano Kit (Agilent Technologies, California, CA, USA). Samples with RIN value and concentration above 8 and 100 ng/µL, respectively, were selected. RNA sequencing was performed by Sequentia Biotech (Barcelona, Spain). The library was prepared using Illumina TruSeq stranded mRNA kit. The library was amplified and paired-ended sequenced using on a NovaSeq 6000 Illumina sequencer (Illumina Inc.; California, CA, USA). Raw data were converted into FASTQ files for posterior analysis.

### 3.6. RNA-Seq Analysis

FASTQ files resulting from RNA-sequencing were processed using the computational resources of the Galician Supercomputational Centre (CESGA). Trimmering was performed using Trimmomatic (version 0.38). The index was generated using Rsem software (version 1.3.1) utilizing Homo sapiens reference genome Ensembl Version GRCh38 and gene transfer format (.gtf) annotation from Ensembl version GRCh38.105. Alignment and count quantification were performed using STAR (version 2.7) and Rsem (version 1.3.1) software, respectively. Differential gene expression was evaluated using DESeq2 R package (version 1.34.0). The Benjamini–Hochberg correction was used to obtain adjusted *p*-values. Shrunken log2 fold changes were obtained using ‘ashr’ shrunkage [87]. Genes with adjusted *p*-value <0.05 and absolute log2 fold change ≥1.5 were considered significant in terms of differential expression. Gene Set Enrichment Analysis was performed using clusterProfiler (version 4.2.2) [88,89], Gene Ontology org.Hs.eg.db, and MSigDB Hallmarks collection gene sets. The Benjamini–Hochberg correction was used to obtain adjusted *p*-values. Gene Ontology over-representation tests for differentially expressed genes were performed using clusterProfiler (version 4.2.2).

### 3.7. Kaplan–Meier Estimates

The Kaplan–Meier plotter online tool was used to correlate gene expression and estimated survival of our candidate synergy genes with the Pan-Can Pancreatic ductal adenocarcinoma subset (n = 177 patients) [90]. Genes with *p*-value < 0.05 and FDR < 0.25 were considered statistically significant.

## 4. Conclusions

In this work, we analyzed the antitumor effect of five drugs that act on epigenetic targets and one drug that restores the p53 function using three different human pancreatic cell lines. In either 2D, 3D, or real-time cell viability assays, we showed that the compounds UVI5008, MS275, and BIX01294 have a good and superior antitumoral efficacy than the rest of the compounds tested, including gemcitabine. P53R3 showed also a good therapeutic efficacy on 3D cell cultures, even in cells that carried a different p53 mutation compared to those targeted by the molecule. The results highlight the need of an ECM and the cell–cell interaction to test the therapeutic efficacy of some drugs that act on cell signaling mechanisms, such as the case of p53 protein signaling.

The combination of UVI5008 or MS275 with gemcitabine can be a good therapeutic alternative with great efficacy against pancreatic tumors that have shown gemcitabine resistance. The therapeutic effect was induced at significant lower doses than the individual treatments, reducing the potential associated chemotherapy toxicity.

The identification of biomarkers of therapeutic effect, such as *GABRQ*, *KCTD4,* or the estrogen pathway signaling, could be useful for evaluating personalized therapies considering the specific mutations or alteration of the epigenetic pattern involved in the tumoral process. Our results open new therapeutic options for PDAC patients and can help in the development of clinical trials that use a combination of these drugs.

## Data Availability

RNA-seq data have been deposited in the ArrayExpress database at EMBL-EBI (www.ebi.ac.uk/arrayexpress) under accession number E-MTAB-11922.

## Supplementary Materials

The following supporting information can be downloaded at: https://www.mdpi.com/article/10.3390/ph15070824/s1, Experimental section and figures of the hemocompatibility studies for UVI5008, MS275, BIX01294, gemcitabine, and the synergistic combinations. Table S1: IC_50_ of the epigenetic inhibitors (1, UVI5008, MS275, psammaplin A, BIX01294), P53R3, and gemcitabine for SKPC-1, MIA PaCa-2, and BxPC-3 cells at 48 h determined by the MTS assay; Table S2: IC_50_ of the epigenetic inhibitors (1, UVI5008, MS275, psammaplin A, BIX01294), P53R3, and gemcitabine for each PDAC cell line at 24, 48, 72, or 96 h, analyzed by xCelligence RTCA system; Table S3: IC_50_ of the epigenetic inhibitors (1, UVI5008, MS275, psammaplin A, BIX01294), P53R3, or gemcitabine in each PDAC cell line at 72 h for the spheroids derived from each PDAC cell line; Table S4: Synergy score by Combenefit software, based on the Bliss Independence model, for different concentrations of UVI5008, MS275, and gemcitabine in the BxPC-3 and MIA PaCa-2 lines; Figure S1: Kinetics of SKPC-1, MIA PaCa-2, and BxPC-3 cell viability incubated with 1 (MS275 analogue), Psammaplin A, and P53R3; Figure S2: Characterization of the hemocompatibility of the epigenetic inhibitors UVI5008, MS275, and BIX01294, gemcitabine (Gem), and the combinations that showed a synergistic antitumor effect (MS275-Gem and UVI5008-Gem); Figure S3: (A) Cluster analysis and heatmap representation of transcripts per million (TPM), row-scaled. (B) Number of differentially expressed (DE) genes shared among treatments when compared to untreated control; Figure S4: GSEA using MSigDB Hallmarks collection; Figure S5: Synergy candidate (SC) genes shared among cell lines and treatments.
