# Supplementary material for: Synergistic Antitumoral Effect of Epigenetic Inhibitors and Gemcitabine in Pancreatic Cancer Cells
Source: Pharmaceuticals (Basel). 2022 Jul 2;15(7):824. doi: 10.3390/ph15070824 (PMC9323654; doi:10.3390/ph15070824)
Supplement: Supplementary file 1 [file pharmaceuticals-15-00824-s001.zip › Supplementary information.pdf]

Synergistic antitumoral effect of epigenetic inhibitors and gemcitabine in pancreatic cancer cells

Immacolata Maietta[1,2†], Amparo Martínez-Pérez[1,2†], Rosana Álvarez [2,3], Ángel R. de Lera[2,3], África González-Fernández[1,2], Rosana Simón[1,2*]

[1] *CINBIO, Universidade de Vigo, Immunology Group, 36310 Vigo, Spain.*

[2]*Instituto de Investigación Sanitaria Galicia Sur (IIS Galicia Sur), SERGAS-UVIGO, Spain.*

[3]*CINBIO, Universidade de Vigo, ORCHID Group, 36310 Vigo, Spain.*

***Corresponding author: Rosana Simón-Vázquez**

rosana.simon@uvigo.es

[†]These authors contributed equally to this work

Materials and methods

Cytocompatibility studies in human peripheral blood mononuclear cells (PBMCs)

PBMCs were isolated from human whole blood samples by using a density gradient centrifugation. The whole blood samples were obtained from healthy volunteers, after obtaining their informed consent, in accordance with approved Institution Review Board protocol (Ref. 2018/369) and the Spanish law. Fresh blood samples were diluted 1:2 in PBS and gently layered over Ficoll-Hypaque (Cytiva, Danaher group, Washington, USA) in Falcon tubes (Corning® Merck, Darmstadt, Germanyu) at a 7:3 blood/Ficoll ratio. The tubes were centrifuged at 2000 rpm for 30 minutes at room temperature and the PBMCs were collected with a Pasteur pipette. After two washing steps with PBS, the PBMCs were counted and diluted at 1×10^6 in RPMI 1640 medium (Corning®), supplemented with 10% fetal bovine serum (FBS, Sigma-Aldrich), 100 U/mL Penicillin, 100 μg/mL Streptomycin, 1μM sodium pyruvate (Gibco, ThermoFisher, Spain). The cells were seeded in 96-well plates and allowed to rest in the cell incubator for 24 hours. PBMCs were treated with compounds UVI5008, MS275 and BIX01294 in combination or not with gemcitabine. Gemcitabine alone was also tested as control. The concentrations used for the individual treatments were 0.5 μM and 1 μM for UVI5008 and for gemcitabine (Gem), and 5 μM and 10 μM for MS275 and BIX01294.

In the combinations, the concentrations tested were: UVI5008 (0.5 μM) + Gem (1 μM); MS275 (5 μM) + Gem (0.5 μM); and MS275 (1 μM) + Gem (0.01 μM). To discard the potential apoptosis or necrosis induced by the treatments, the cells were labelled with AnexinV/propidium iodide (PI) and measured in the flow cytometer. For that, the cells were detached from the plates with trypsin, washed with PBS and diluted in 100 μL Annexin V Binding Buffer (Immunostep, Spain). Subsequently, 3 μL Annexin V-FITC (Immunostep, Spain) and 3 μL of PI at 250 μg/mL (AnaSpec, California, USA) were added to each sample. The cells were incubated with AnexinV/PI for 15 minutes and analysed on a BD Accuri™ C6 Flow Cytometer (BD Biosciences, USA) using the software provided with the equipment.

Haemolysis

To analyse the potential haemolysis induced by the different compounds, approximately 5 mL of blood from an EDTA K2 tube (Vacutainer®, BD Bioscience, USA) was diluted 1:2 with PBS and centrifuged at 2000 rpm for 10 minutes at 4 ºC. After discarding the supernatant, the pellet was weighed and diluted at a concentration of 3% (w/v) in PBS. Subsequently, 80 μL of the diluted blood suspension was plated in the 96-well round (U) bottom plate and treated with 80 μL of UVI5008, MS275, BIX01294 alone or in combination with gemcitabine at the same concentrations described above. 0.1% Triton was used as positive control. The samples were incubated for 4 hours at 37 ºC and centrifuged at 2000 rpm for 10 minutes at 4 ºC. An aliquot of 80 μL of the blood-treatment suspension was taken and placed into a plate with a 96-well flat bottom plate. The absorbance was, subsequently, read at 570 nm. Haemolysis, expressed as a percentage, was calculated as follows:

% Haemolysis = ([A]sample treated - [A]control / [A]positive control - [A]control) × 100

where: [A]sample treated is the absorbance of the cells incubated with the drugs, [A]control is the absorbance of the PBS and [A]positive control is the absorbance of the haemolytic sample treated with 0.1% Triton.

Tables

Table S1. IC_{50} of the epigenetic inhibitors (1, UVI5008, MS275, psammaplin A, BIX01294), P53R3 and gemcitabine for SKPC-1, MIA PaCa-2 and BxPC-3 cells at 48 hours determined by the MTS assay.

IC_{50} (µM)	SKPC-1	MIA PaCa-2	BxPC-3
**1**	**15**	+50	+50
**UVI5008**	**1.7**	**1**	**1.7**
**MS275**	**14**	18	**14.9**
**psammaplin A**	19.7	**12**	**7.9**
**BIX01294**	**11.6**	**2**	**12**
**P53R3**	+50	29	31.6
**Gemcitabine**	21	+50	**2**

$IC_{50} \leq 15$ µM are shown in bold

Table S2. IC_{50} of the epigenetic inhibitors (1, UVI5008, MS275, psammaplin A, BIX01294), P53R3 and gemcitabine for each PDAC cell line at 24, 48, 72 or 96 hours, analysed by xCelligence RTCA system.

IC_{50} (µM)	SKPC-1				MIA PaCa-2				BxPC-3			
	24h	48h	72h	96h	24h	48h	72h	96h	24h	48h	72h	96h
**1**	32	20	18	18	45	**12**	**8**	**7**				
**UVI5008**	**3**	**1.4**	**2**	**2**	**9**	**0.5**	**0.3**	**0.2**	**9.8**	**2**	**0.8**	**0.7**
**MS275**	**14**	**4.5**	**3**	**3**	50	**3**	**2**	**2**	**10**	**10**	**10**	**9,8**
**psammaplin A**	**9**	**9**	**10**	**11**	23	**4**	**2**	**2**				
**BIX01294**	**6**	**8**	**7**	**6**	**7**	**5**	**4**	**5**				
**P53R3**	+50	**14**	**12**	**12**	37	19	16	17				
**Gemcitabine**	+50	+50	**<1**	**1**	+50	**9**	**<1**	**<1**	31	**2**	**0.4**	**<1**

$IC_{50} \leq 15$ µM are shown in bold. Squares in white (not tested).

Table S3. IC_{50} of the epigenetic inhibitors (1, UVI5008, MS275, psammaplin A, BIX01294), P53R3 or gemcitabine in each PDAC cell line at 72 hours for the spheroids derived from each PDAC cell line.

**IC_{50} (µM)**	**SKPC-1**	**MIA PaCa-2**	**BxPC-3**
**1**	**5**	25	40
**UVI5008**	**7**	**6**	**2**
**MS275**	**<1**	+50	**5**
**psammaplin A**	**15**	**10**	**6**
**BIX01294**	**<1**	**6**	**<1**
**P53R3**	**15**	**12**	**13**
**Gemcitabine**	20	+50	**8**

$IC_{50} \leq 15$ µM are shown in bold.

Table S4. Synergy score by Combenefit software, based on Bliss Independence model, for different concentrations of UVI5008, MS275 and gemcitabine in the BxPC-3 and MIA PaCa-2 lines.

Cell line	Gem (µM)	MS275 (µM)	Syn score	Interpretation
MIAPaCa-2	0.5	1	17	Additive
	0.5	2.5	25	Synergism
	**0.5**	**5**	**40**	**Synergism**
	1	1	17	Additive
	1	2.5	25	Synergism
	1	5	40	Synergism
	2.5	1	8	Additive
	2.5	2.5	18	Additive
	2.5	5	32	Synergism
BxPC-3	**0.01**	**1**	**37**	**Synergism**
	0.01	2.5	30	Synergism
	0.01	5	26	Synergism
	0.05	1	15	Additive
	0.05	2.5	13	Additive
	0.05	5	12	Additive
	0.1	1	18	Additive
	0.1	2.5	10	Additive
	0.1	5	8	Additive
**Cell line**	**Gem (µM)**	**UVI5008 (µM)**	**Syn score**	**Interpretation**

MIAPaCa-2	0.5	0.2	-3	Additive
	0.5	0.5	25	Synergism
	0.5	1	29	Synergism
	1	0.2	16	Additive
	**1**	**0.5**	**44**	**Synergism**
	1	1	48	Synergism
	2.5	0.2	8	Additive
	2.5	0.5	43	Synergism
	2.5	1	46	Synergism

Figures

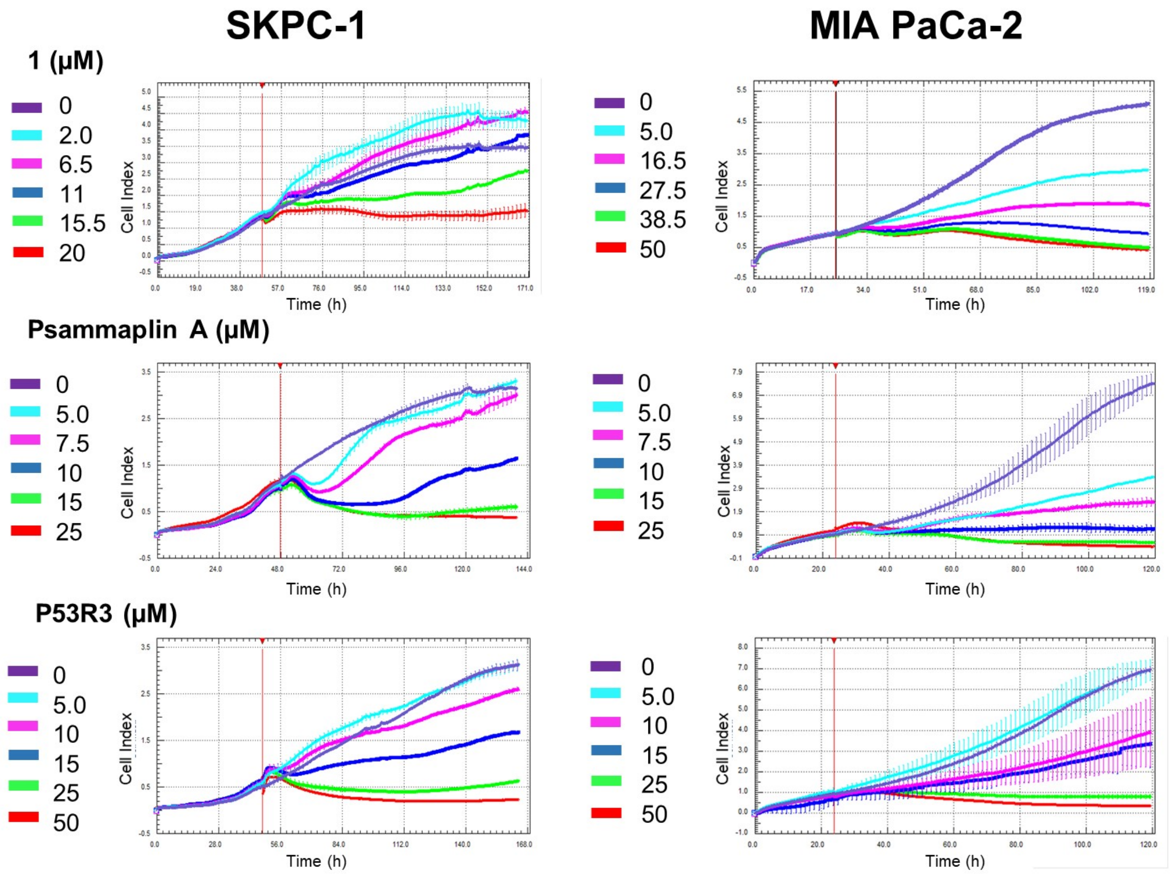

Figure S1. Kinetics of SKPC-1, MIA PaCa-2 and BxPC-3 cell viability incubated with 1 (MS275 analogue), Psammaplin A, and P53R3. The treatments were added after cell stabilization (vertical red lines).

A

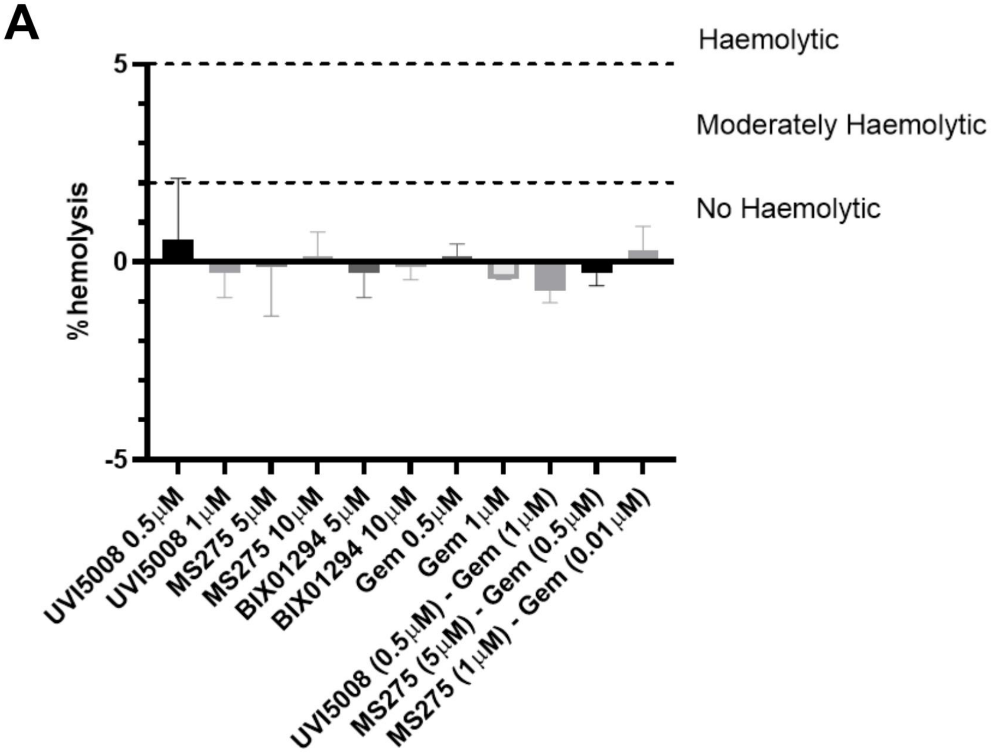

B

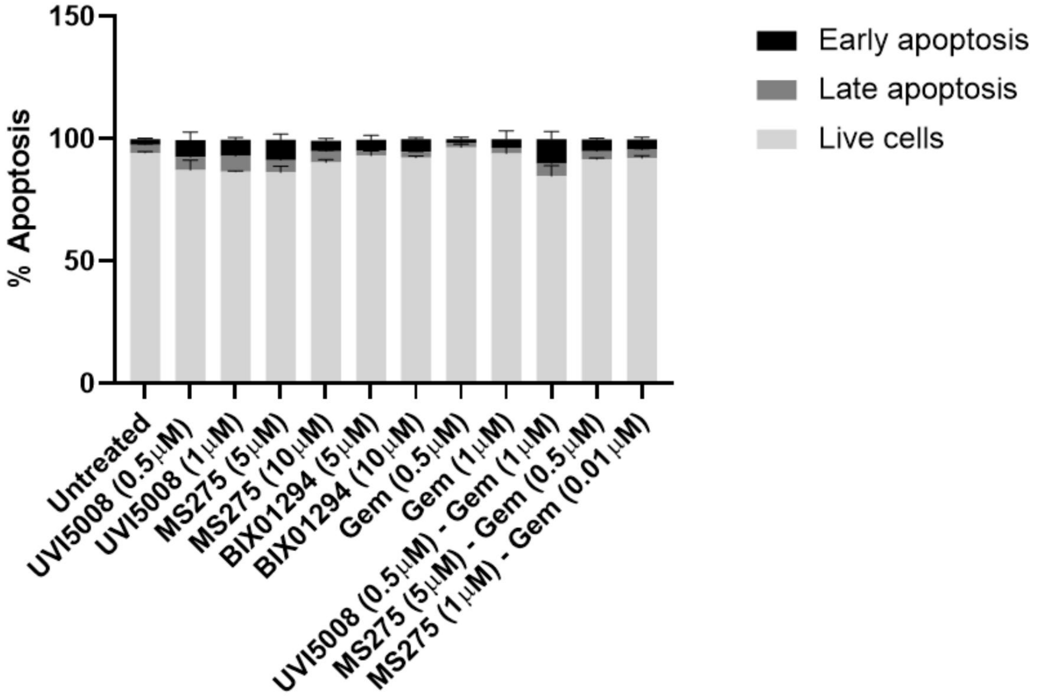

Figure S2. Characterization of the hemocompatibility of the epigenetic inhibitors UVI5008, MS275 and BIX01294, gemcitabine (Gem) and the combinations that showed a synergistic antitumor effect (MS275-Gem and UVI5008-Gem). **A**. Hemolysis. **B-D**. Percentage of live, early apoptotic and late apoptotic cell populations in PBMCs

incubated with the individual compounds. The concentrations tested were those that induced a significant decrease in the viability of the PDAC cell lines.

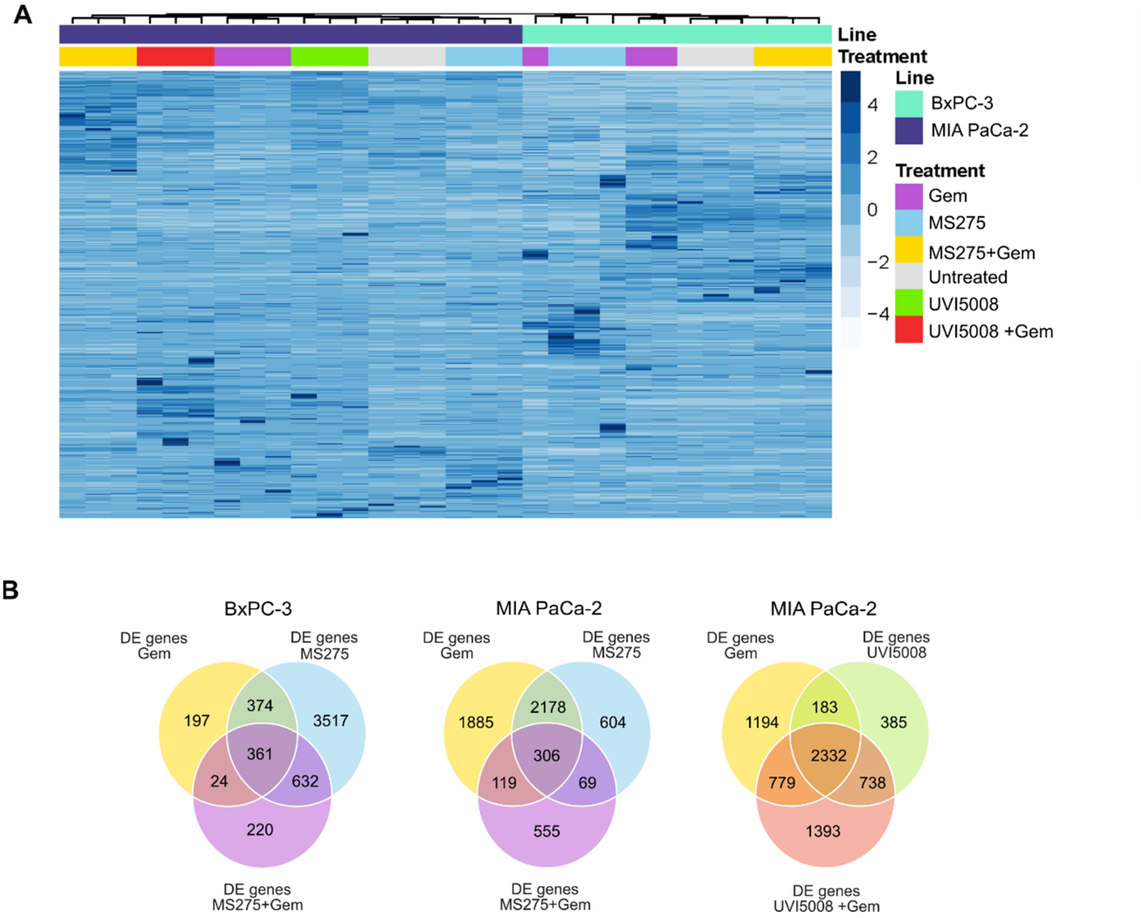

Figure S3. (**A**) Cluster analysis and heatmap representation of transcripts per million (TPM), row-scaled. (**B**) Number of differentially expressed (DE) genes shared among treatments when compared to untreated control.

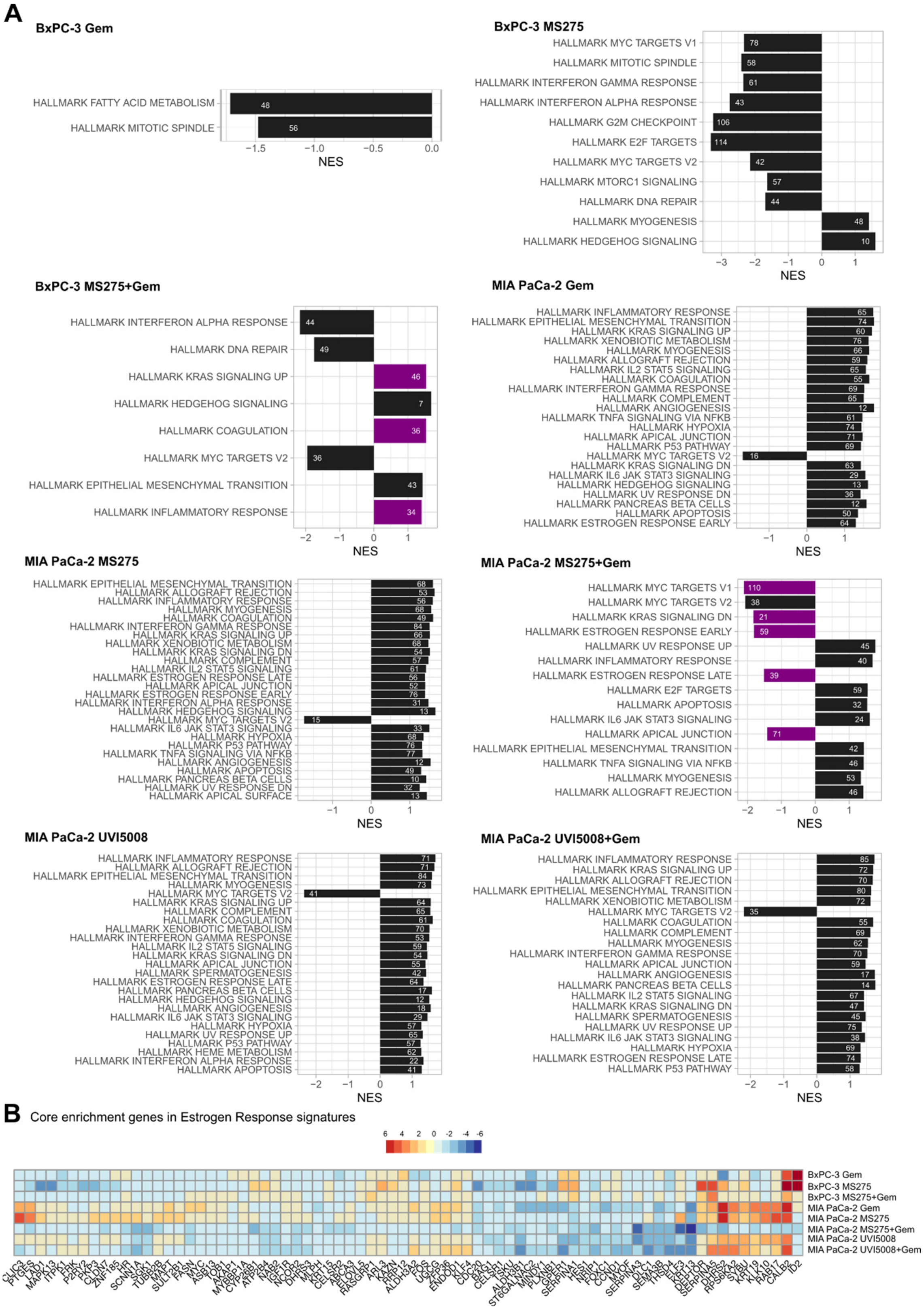

Figure S4. GSEA using MSigDB Hallmarks collection. (**A**) Gene set enrichment analysis compared the treatment-induced transcriptomes with published molecular signatures in the MSigDB library. Unique signatures reported in the combinatory treatment UVI5008+Gem and MS275+Gem that were not found in the respective single treatments are colored. Signature items are ordered by p-adjusted. (**B**) Genes in the core enrichment

of Early Estrogen Response and Late Estrogen Response sets enriched in MIA PaCa-2 MS275+Gem signature. Genes are colored by their expression levels. *NES: normalized enrichment score.*

A

	BxPC-3 MS275+Gem SC	MIA PaCa-2 MS275+Gem SC	MIA PaCa-2 UVI5008+Gem SC
lncRNA	43	185	343
misc_RNA	NA	1	1
nonsense_mediated_decay	12	23	57
processed_pseudogene	3	18	72
processed_transcript	19	47	109
protein_coding	139	355	715
retained_intron	6	23	51
scRNA	NA	1	NA
TEC	1	5	18
TR_V_gene	NA	NA	5
transcribed_processed_pseudogene	NA	1	2
transcribed_unprocessed_pseudogene	3	NA	6
unitary_pseudogene	NA	1	1
unprocessed_pseudogene	NA	2	16

B

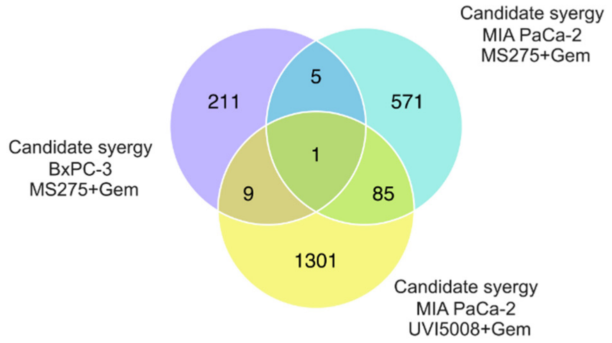

C

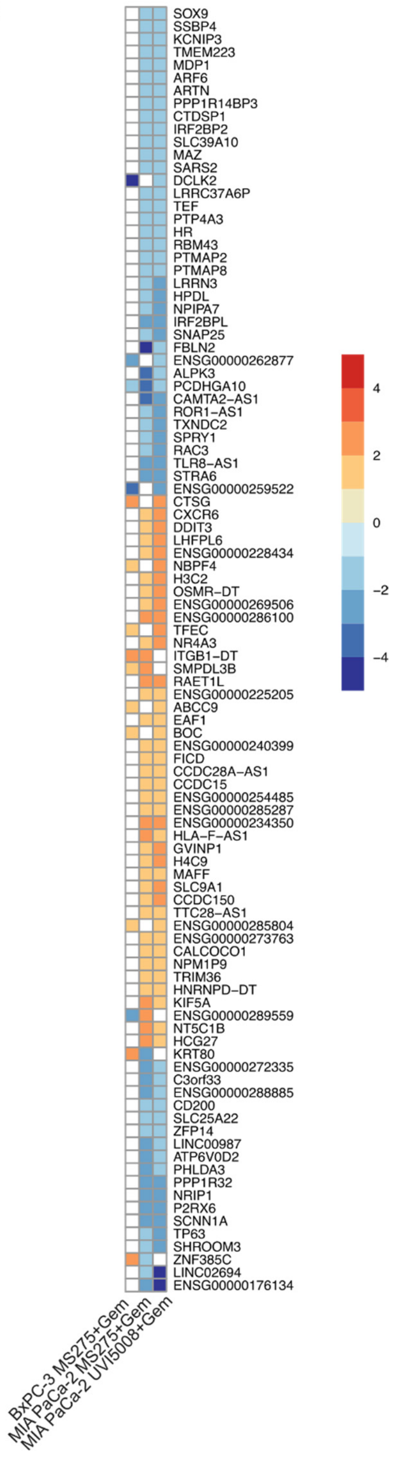

Figure S5. Synergy candidate (SC) genes shared among cell lines and treatments. (**A**) Biotype of the SC genes. (**B**) Number of SC genes shared among BxPC-3 MS275+Gem, MIA PaCa-2 MS275+Gem and MIA PaCa-2 UVI5008+Gem. (**C**) Heatmap

representation of SC genes shared among cell lines and treatments. Genes are colored by their expression levels. *NA* values are colored in white.

Supplementary Figures S6-S12. Kaplan-Meier estimates of SC gene expression in PDAC patients. It is depicted the down or upregulated SC genes found in BxPC3 (MS275+Gem), MIA PaCa-2 (MS275+Gem) and MIA PaCa-2 (UVI5008+Gem) whose low or high expression, respectively, has been associated to statistically improved survival in PDAC patients.
