# Supplementary figures and images for: Synergistic Antitumoral Effect of Epigenetic Inhibitors and Gemcitabine in Pancreatic Cancer Cells
Source: Pharmaceuticals (Basel). 2022 Jul 2;15(7):824. doi: 10.3390/ph15070824 (PMC9323654; doi:10.3390/ph15070824)

# MIA PaCa-2 UVI5008+Gem

## Upregulated - Part 2

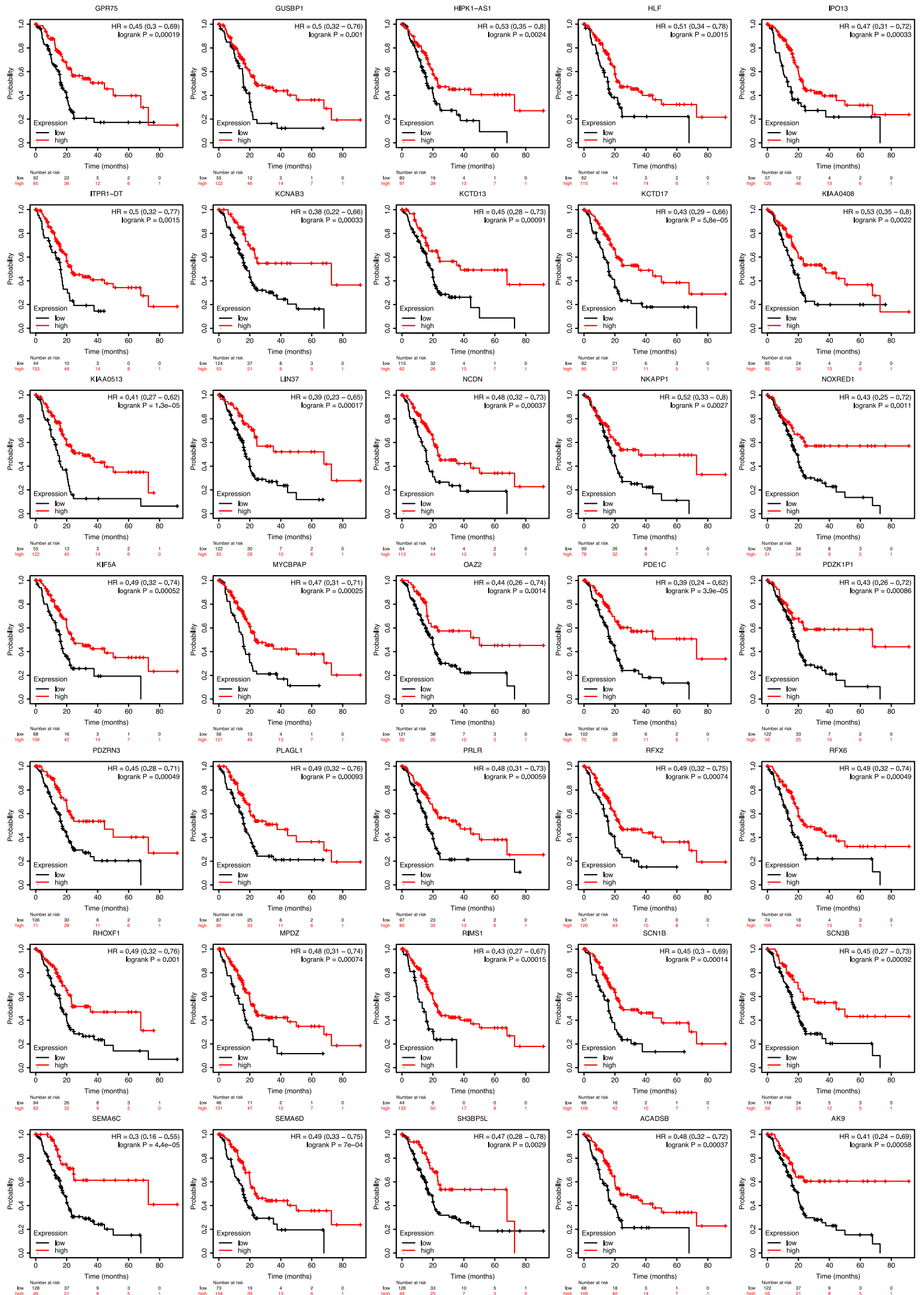

Supplement: Supplementary file 1 [file pharmaceuticals-15-00824-s001.zip › Supplementary Figure S10.pdf]

# MIA PaCa-2 UVI5008+Gem

## Upregulated - Part 3

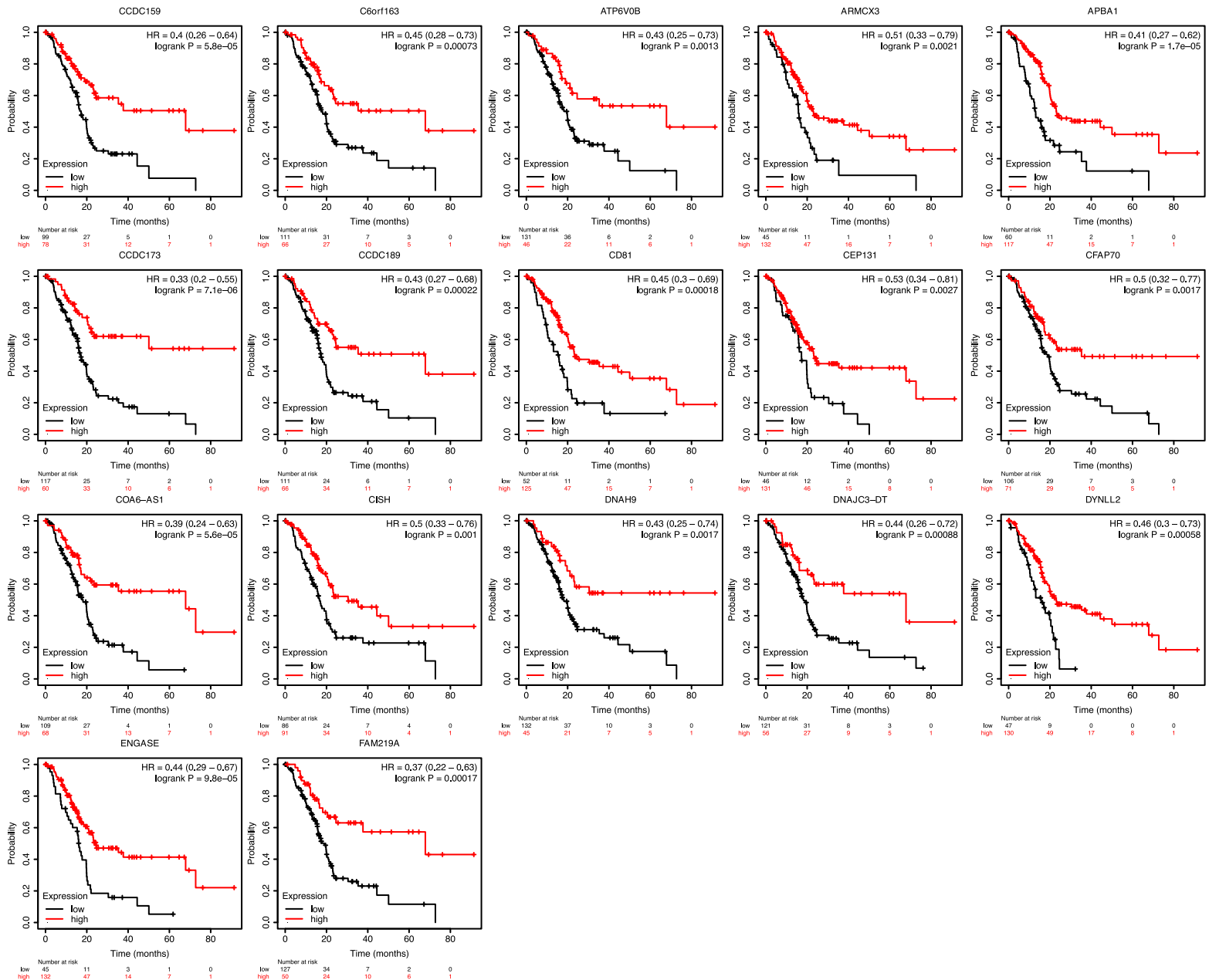

## Downregulated

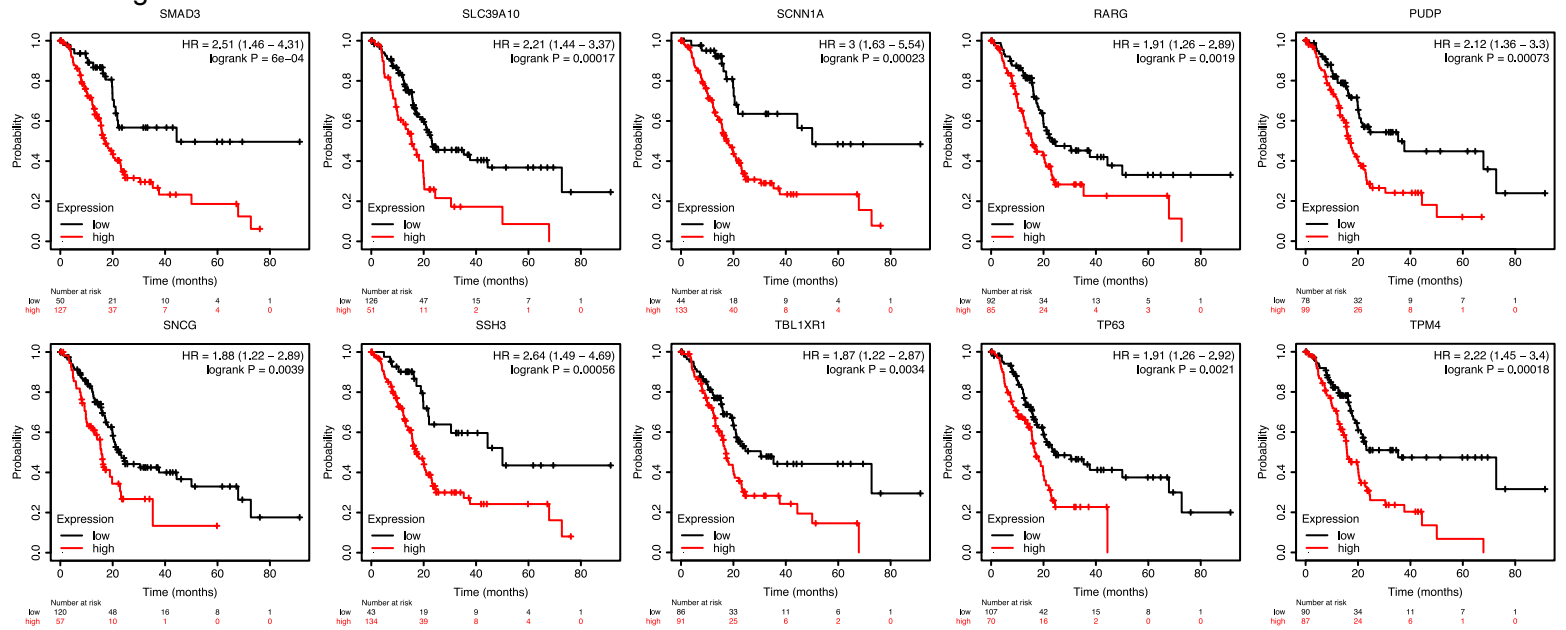

Supplement: Supplementary file 1 [file pharmaceuticals-15-00824-s001.zip › Supplementary Figure S11.pdf]

MIA PaCa-2 UVI5008+Gem  
Downregulated - Part 2

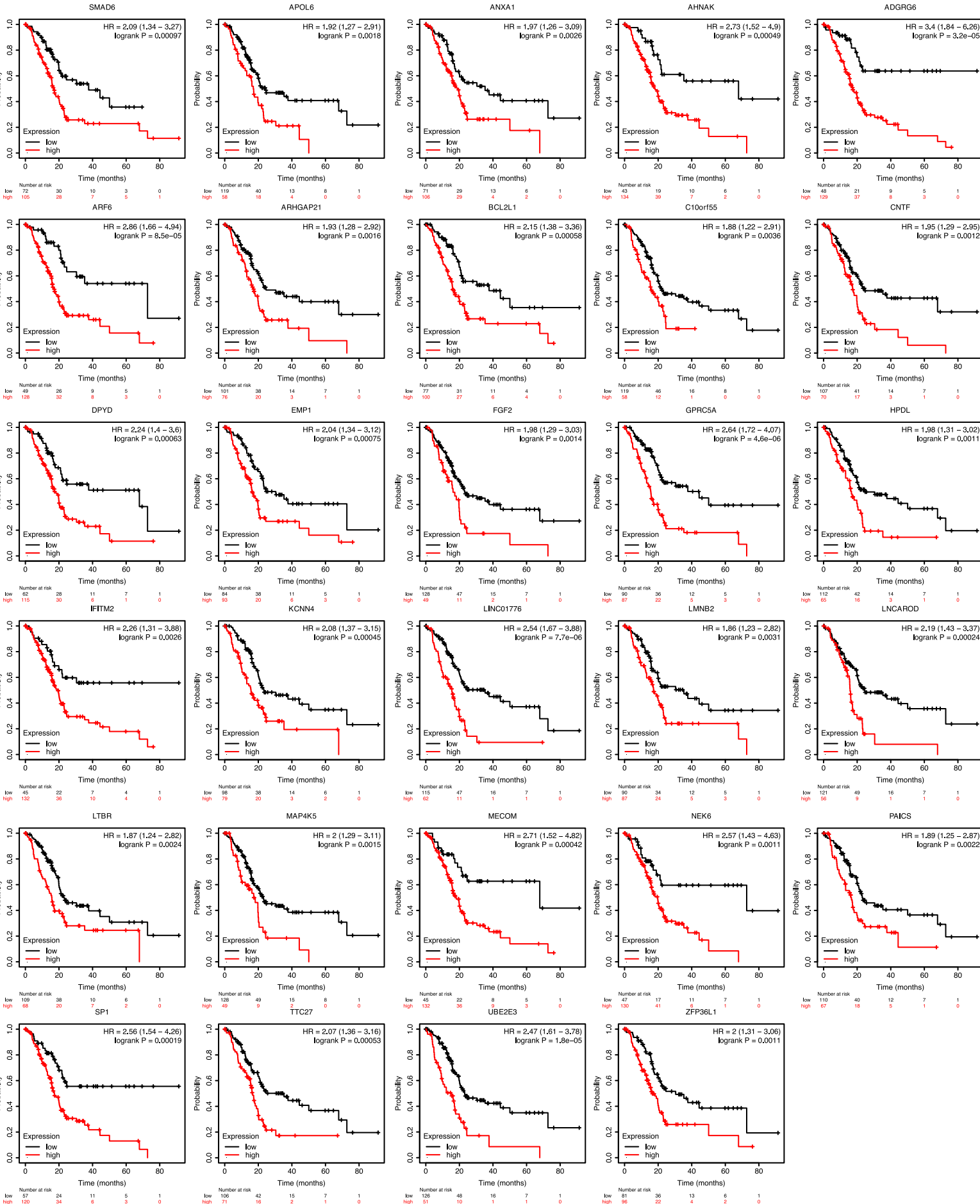

Supplement: Supplementary file 1 [file pharmaceuticals-15-00824-s001.zip › Supplementary Figure S12.pdf]

BxPC-3 MS275+Gem  
Upregulated

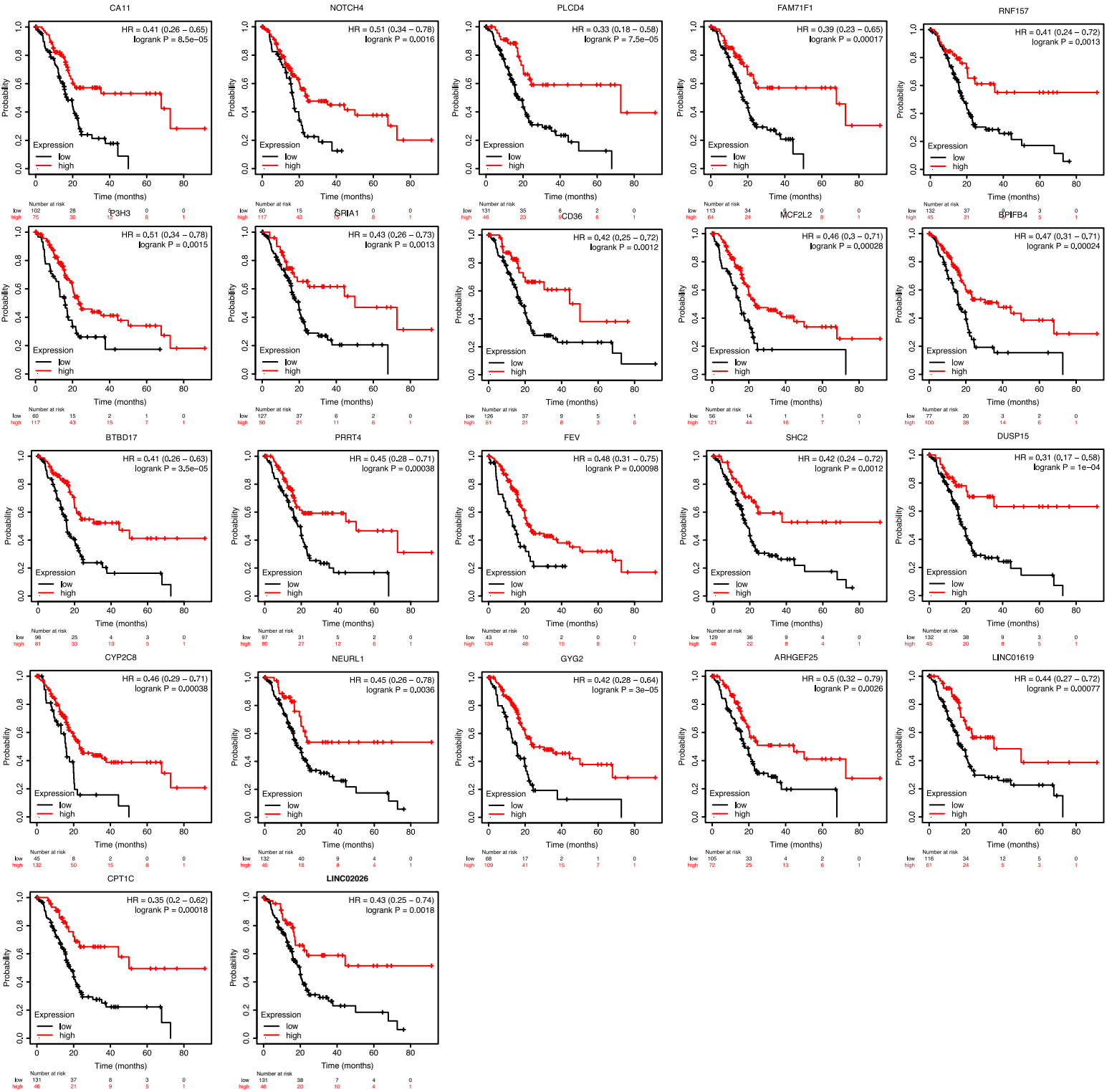

Downregulated

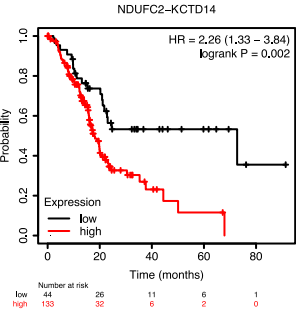

Supplement: Supplementary file 1 [file pharmaceuticals-15-00824-s001.zip › Supplementary Figure S6.pdf]

# MIA PaCa-2 MS275+Gem

## Upregulated

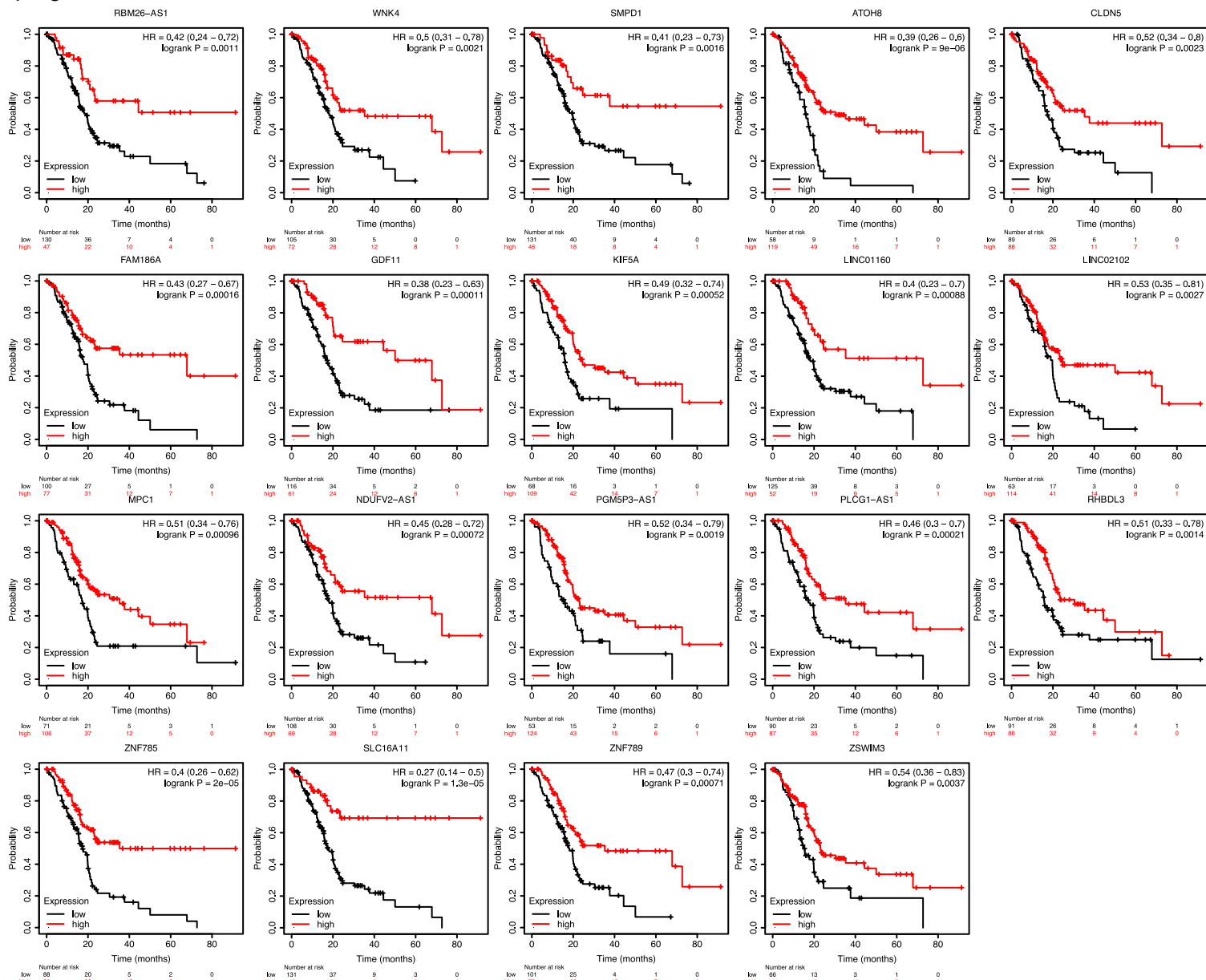

## Downregulated

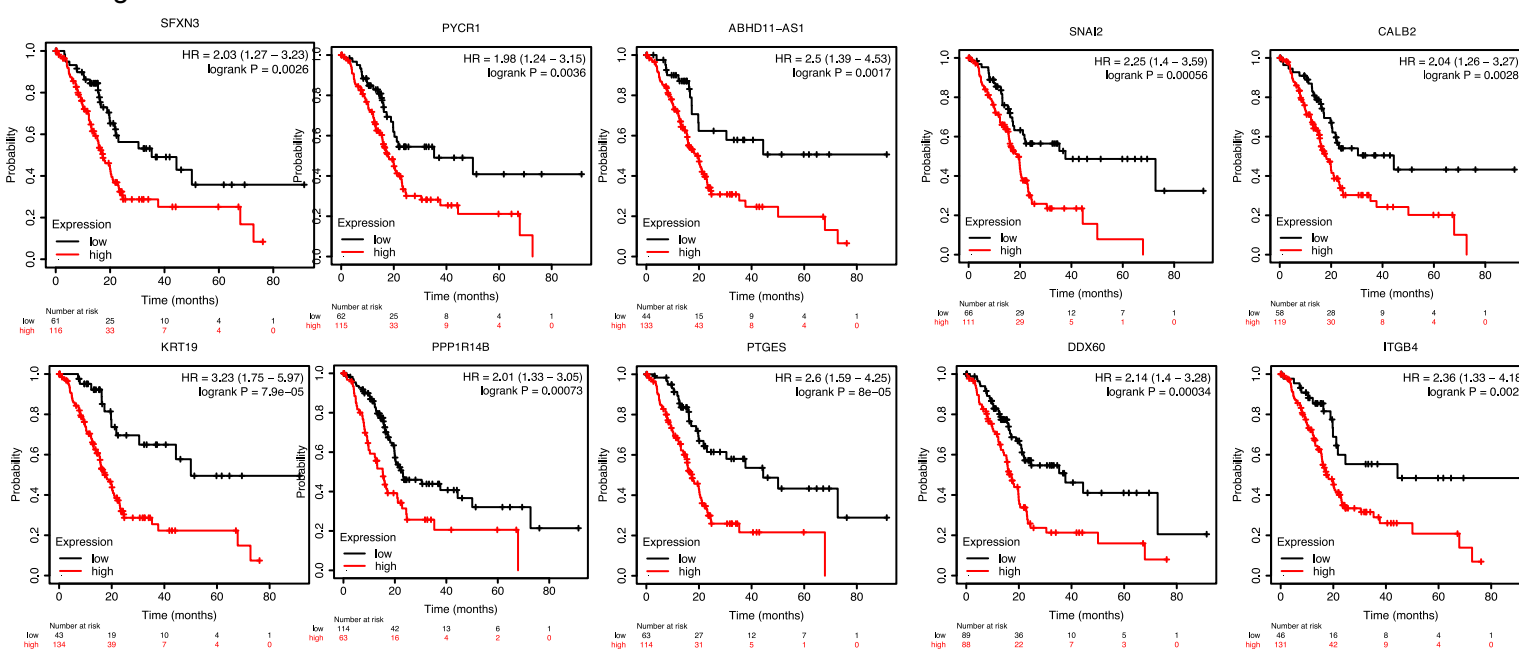

Supplement: Supplementary file 1 [file pharmaceuticals-15-00824-s001.zip › Supplementary Figure S7.pdf]

# MIA PaCa-2 MS275+Gem

## Downregulated (Part 2)

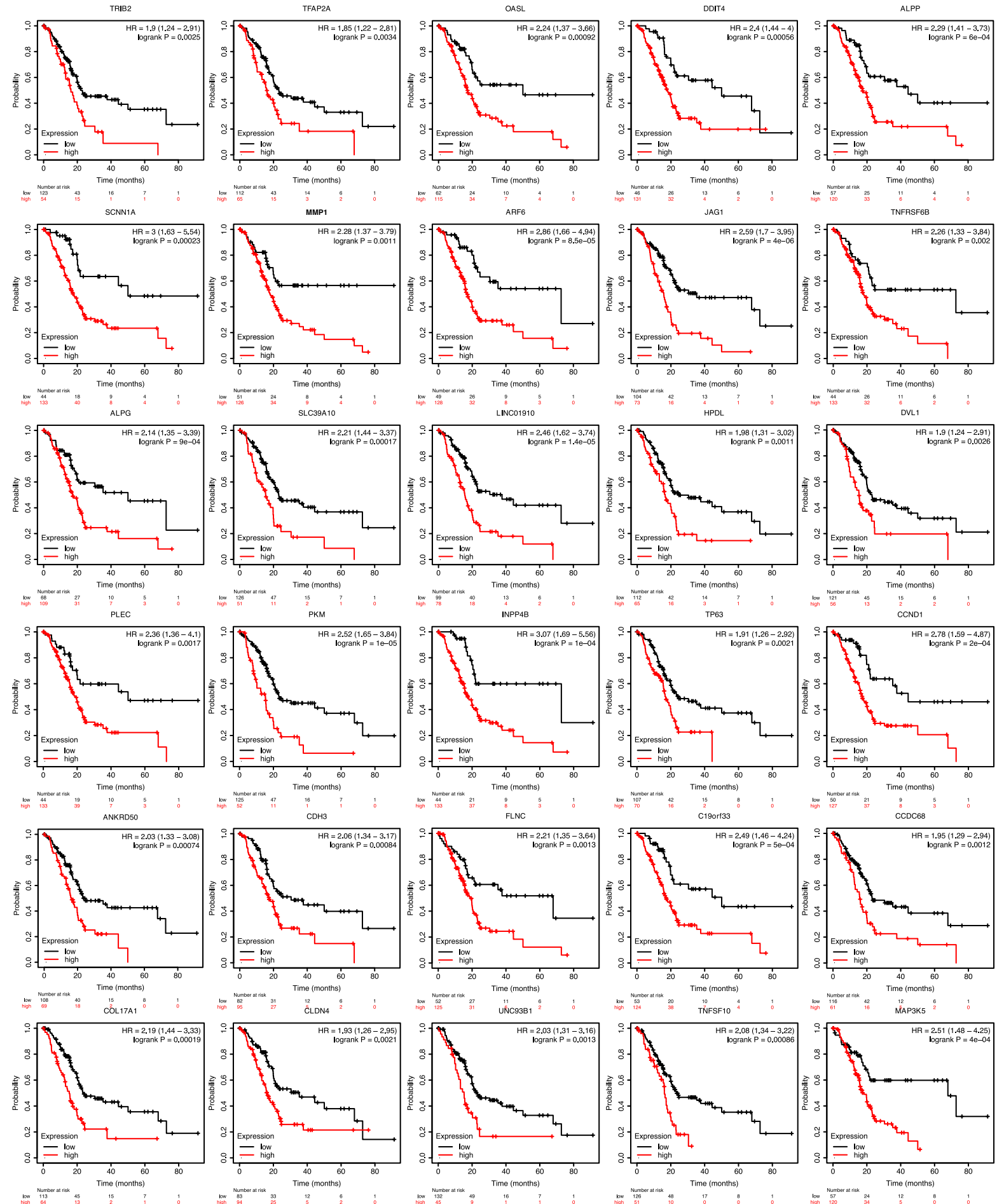

Supplement: Supplementary file 1 [file pharmaceuticals-15-00824-s001.zip › Supplementary Figure S8.pdf]

# MIA PaCa-2 UVI5008+Gem Upregulated

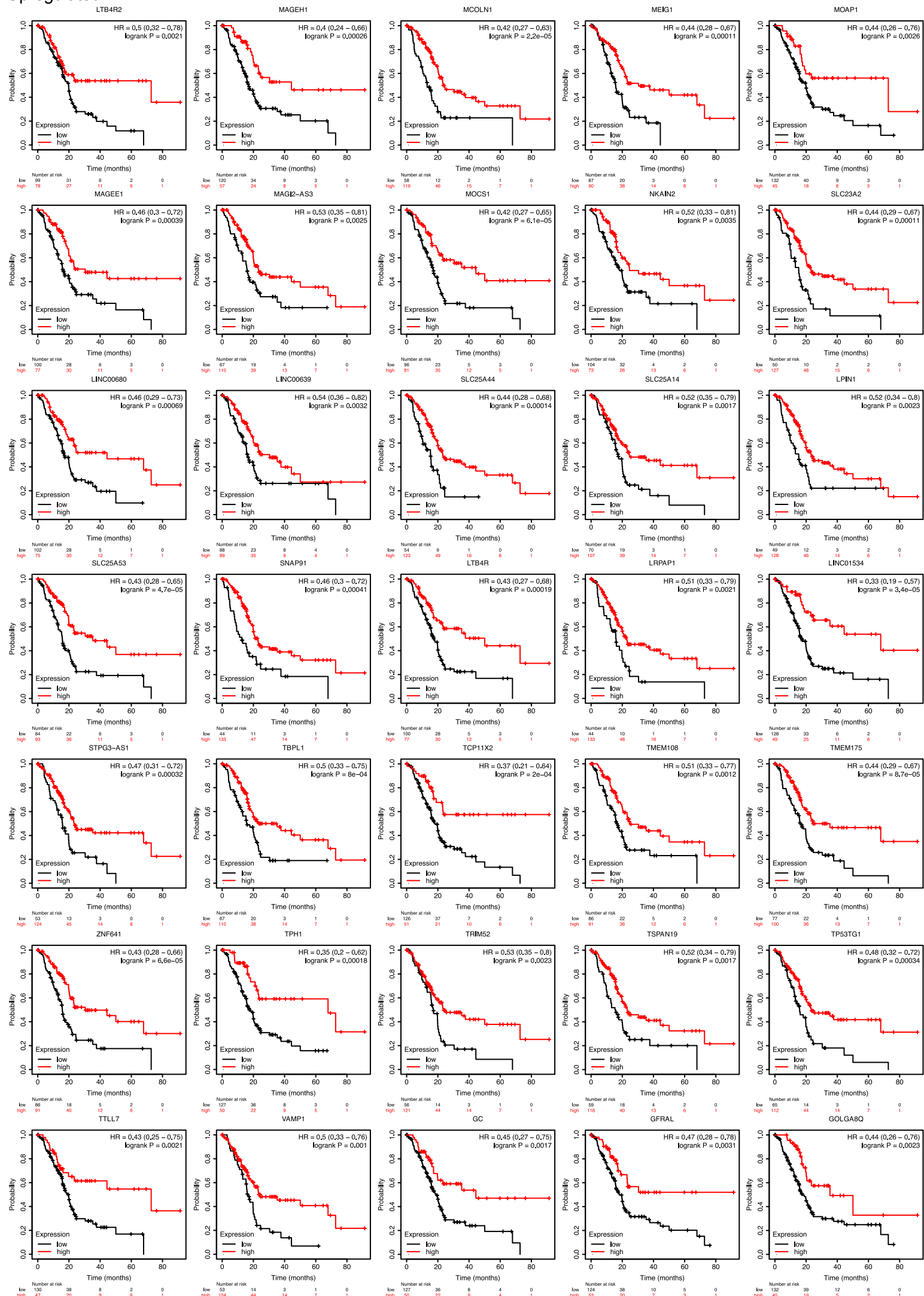

Supplement: Supplementary file 1 [file pharmaceuticals-15-00824-s001.zip › Supplementary Figure S9.pdf]
